# Supplementary figures and images for: Relationships between climate and phylogenetic community structure of fossil pollen assemblages are not constant during the last deglaciation
Source: PLoS One. 2021 Jul 8;16(7):e0240957. doi: 10.1371/journal.pone.0240957 (PMC8266067; doi:10.1371/journal.pone.0240957)

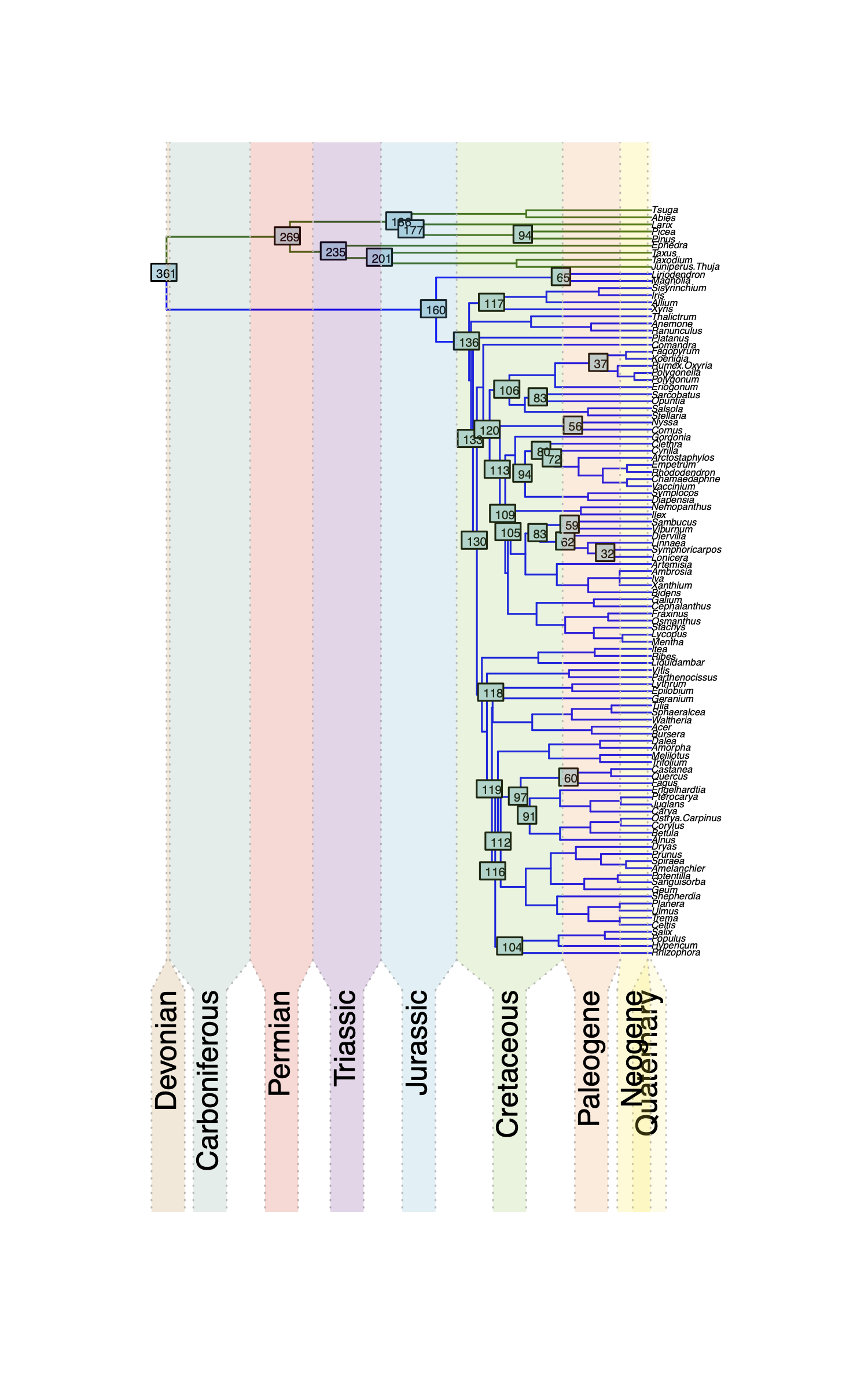

Supplement: S1 Fig — The tree is pruned to the taxa in the fossil pollen dataset. Node labels indicate estimated age of the node as in the ‘datelife’ database. Ages for the rest of the nodes were estimated using the bladj algorithm as implemented in the ‘picante’ package in R. (TIFF) [file pone.0240957.s001.tiff]

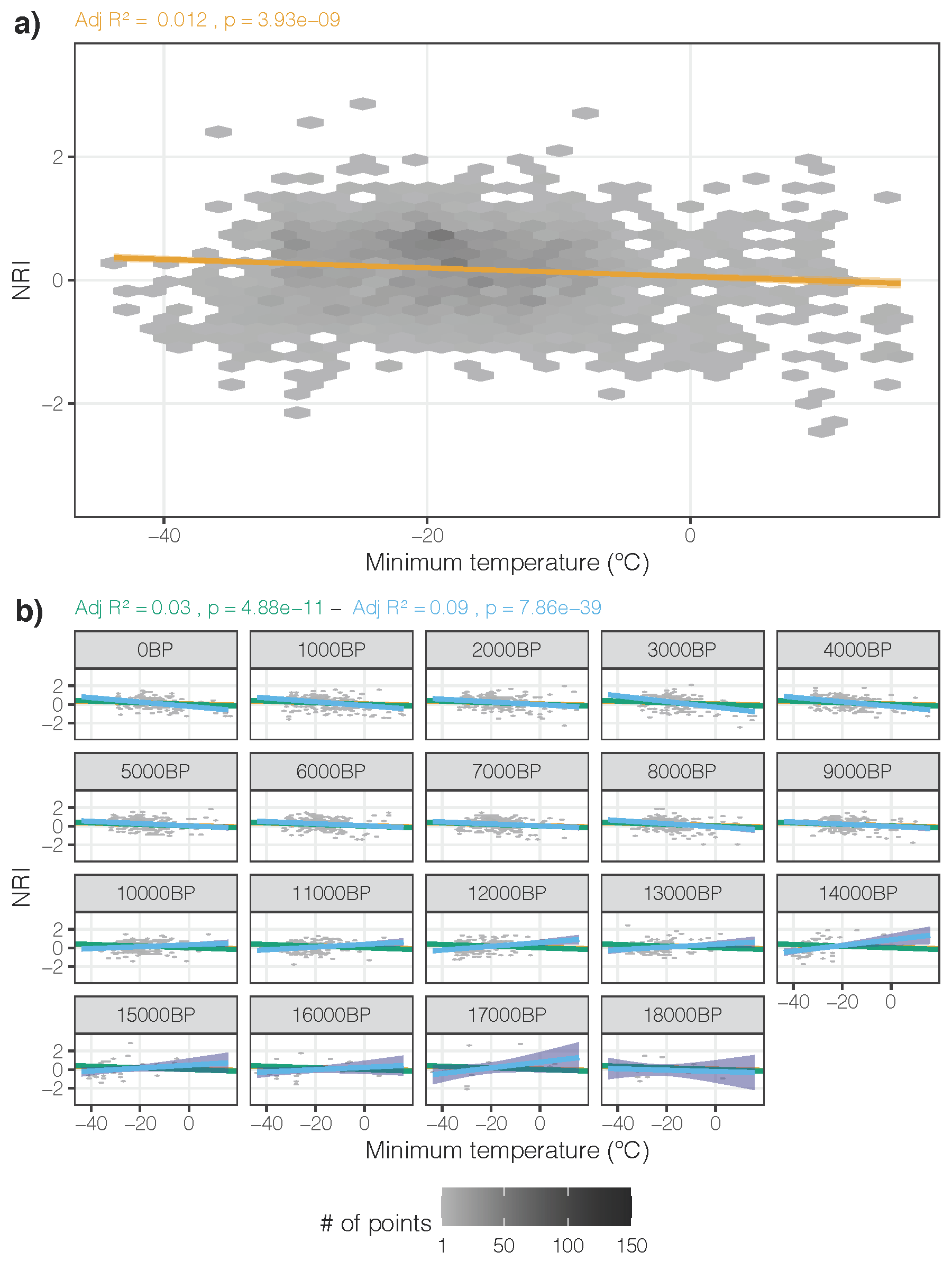

Supplement: S2 Fig — Stable-Relationship (orange line), Stable-Slope (green line) and Changed-Relationship (blue line). Tmin = minimum temperature of the coldest month; Tmax = maximum temperature of the warmest month; Pmin = minimum precipitation of the driest month; Pmax = maximum precipitation of the wettest month; AET = mean yearly actual evapotranspiration; ETR = mean yearly ratio of actual and potential evapotranspiration; WDI = mean yearly water deficit index; DEGLAC = time-since-deglaciation. (ZIP) [file pone.0240957.s002.zip › S2_Figure_Page_1.tiff]

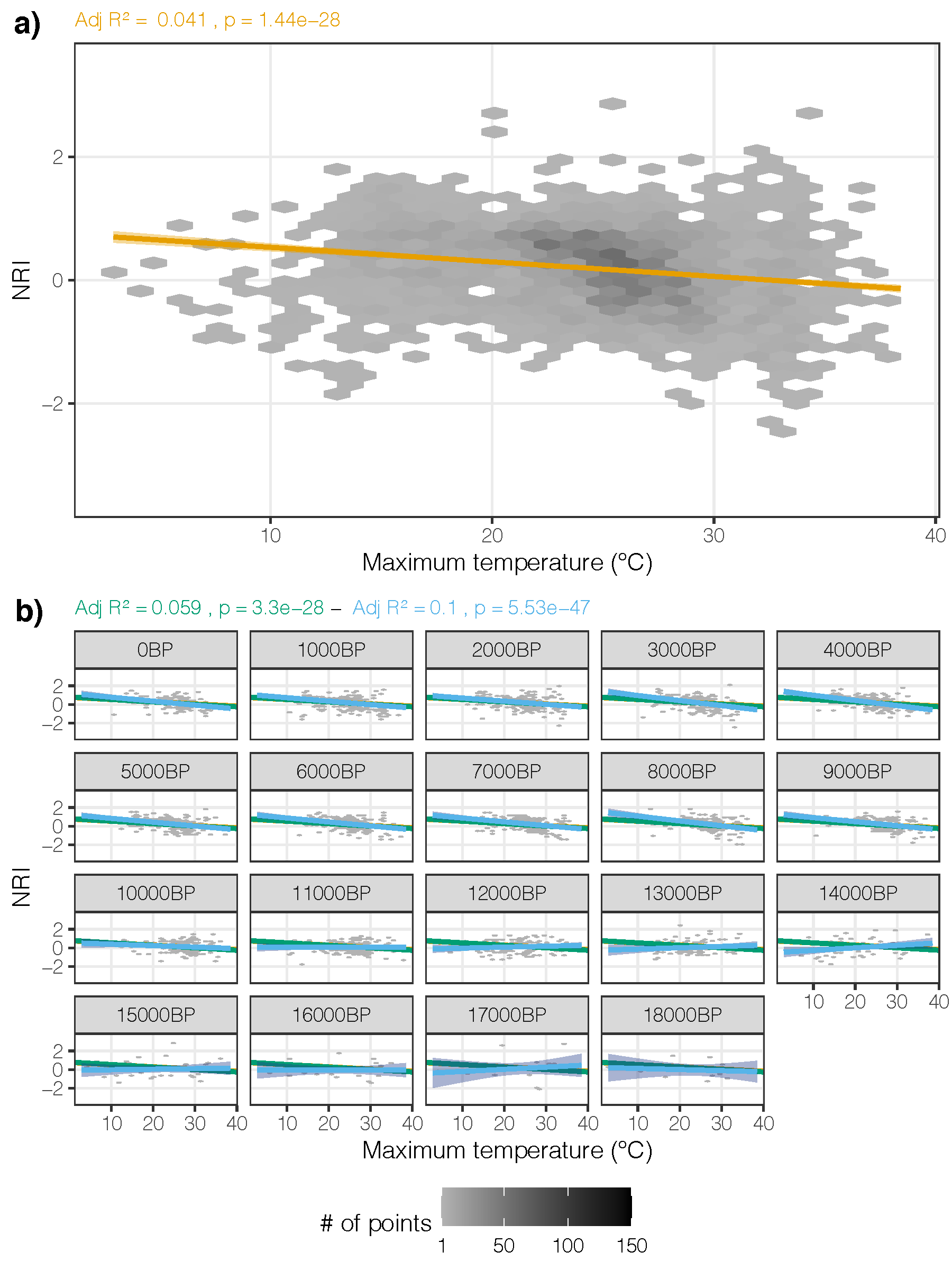

Supplement: S2 Fig — Stable-Relationship (orange line), Stable-Slope (green line) and Changed-Relationship (blue line). Tmin = minimum temperature of the coldest month; Tmax = maximum temperature of the warmest month; Pmin = minimum precipitation of the driest month; Pmax = maximum precipitation of the wettest month; AET = mean yearly actual evapotranspiration; ETR = mean yearly ratio of actual and potential evapotranspiration; WDI = mean yearly water deficit index; DEGLAC = time-since-deglaciation. (ZIP) [file pone.0240957.s002.zip › S2_Figure_Page_2.tiff]

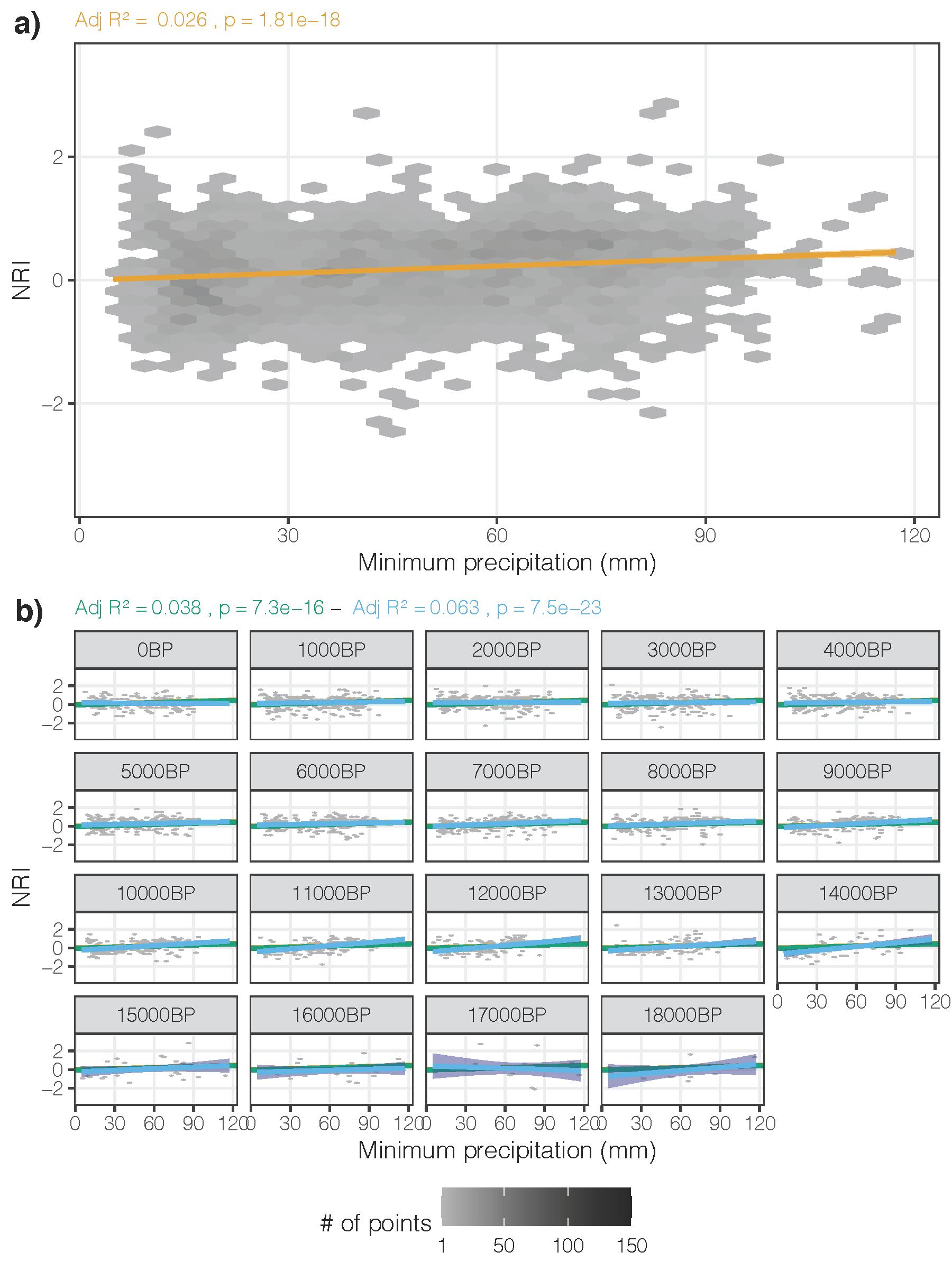

Supplement: S2 Fig — Stable-Relationship (orange line), Stable-Slope (green line) and Changed-Relationship (blue line). Tmin = minimum temperature of the coldest month; Tmax = maximum temperature of the warmest month; Pmin = minimum precipitation of the driest month; Pmax = maximum precipitation of the wettest month; AET = mean yearly actual evapotranspiration; ETR = mean yearly ratio of actual and potential evapotranspiration; WDI = mean yearly water deficit index; DEGLAC = time-since-deglaciation. (ZIP) [file pone.0240957.s002.zip › S2_Figure_Page_3.tiff]

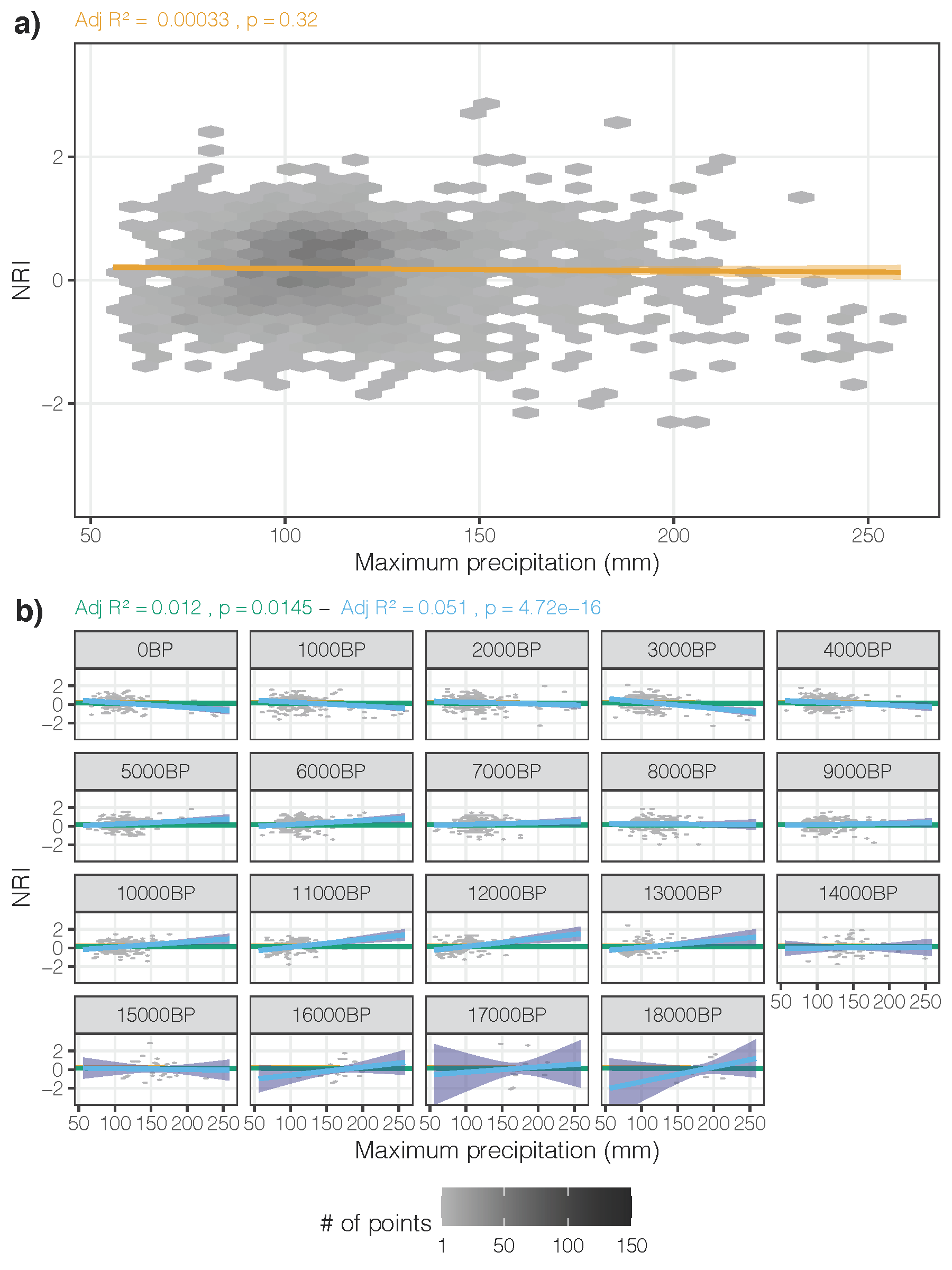

Supplement: S2 Fig — Stable-Relationship (orange line), Stable-Slope (green line) and Changed-Relationship (blue line). Tmin = minimum temperature of the coldest month; Tmax = maximum temperature of the warmest month; Pmin = minimum precipitation of the driest month; Pmax = maximum precipitation of the wettest month; AET = mean yearly actual evapotranspiration; ETR = mean yearly ratio of actual and potential evapotranspiration; WDI = mean yearly water deficit index; DEGLAC = time-since-deglaciation. (ZIP) [file pone.0240957.s002.zip › S2_Figure_Page_4.tiff]

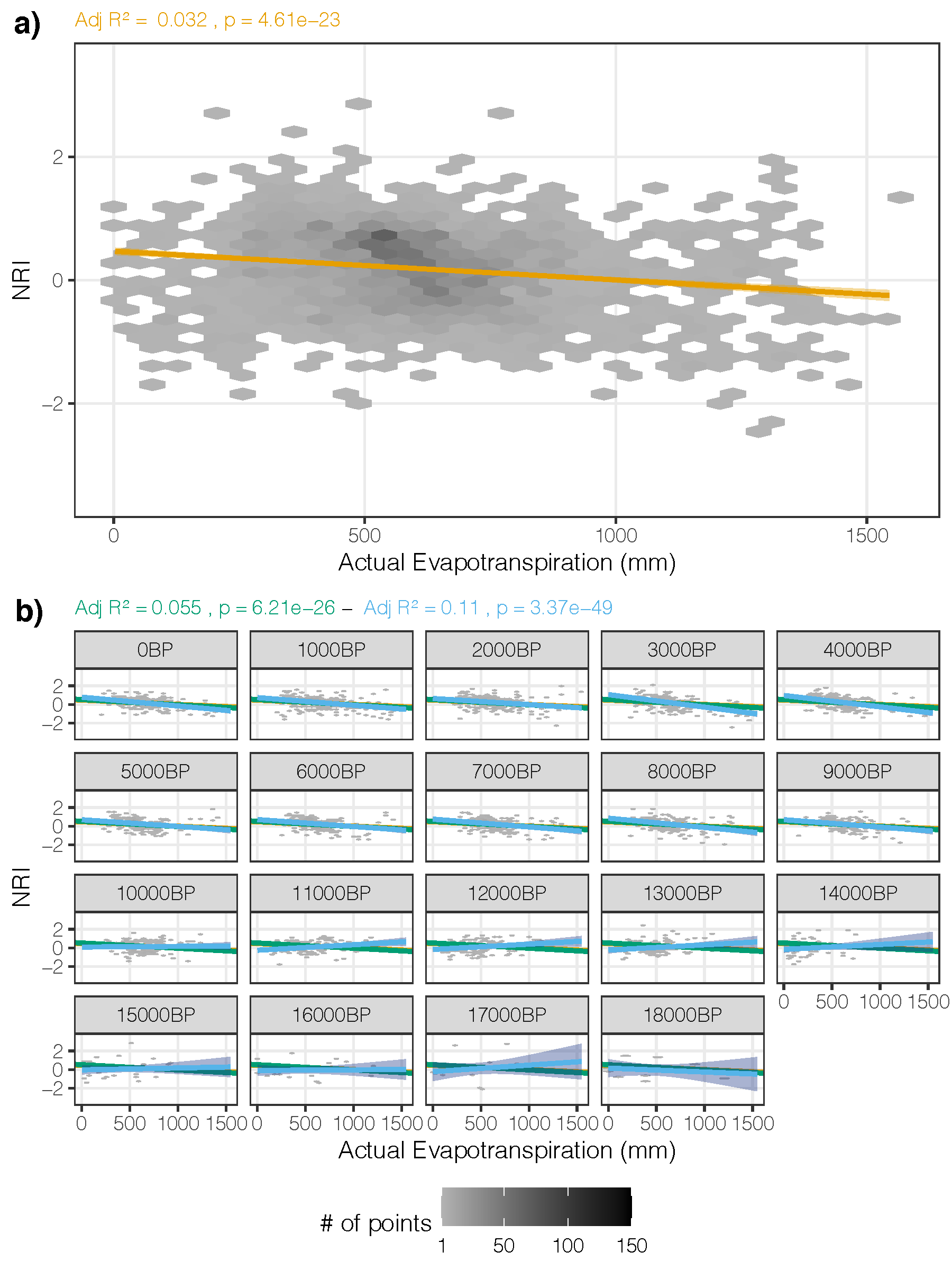

Supplement: S2 Fig — Stable-Relationship (orange line), Stable-Slope (green line) and Changed-Relationship (blue line). Tmin = minimum temperature of the coldest month; Tmax = maximum temperature of the warmest month; Pmin = minimum precipitation of the driest month; Pmax = maximum precipitation of the wettest month; AET = mean yearly actual evapotranspiration; ETR = mean yearly ratio of actual and potential evapotranspiration; WDI = mean yearly water deficit index; DEGLAC = time-since-deglaciation. (ZIP) [file pone.0240957.s002.zip › S2_Figure_Page_5.tiff]

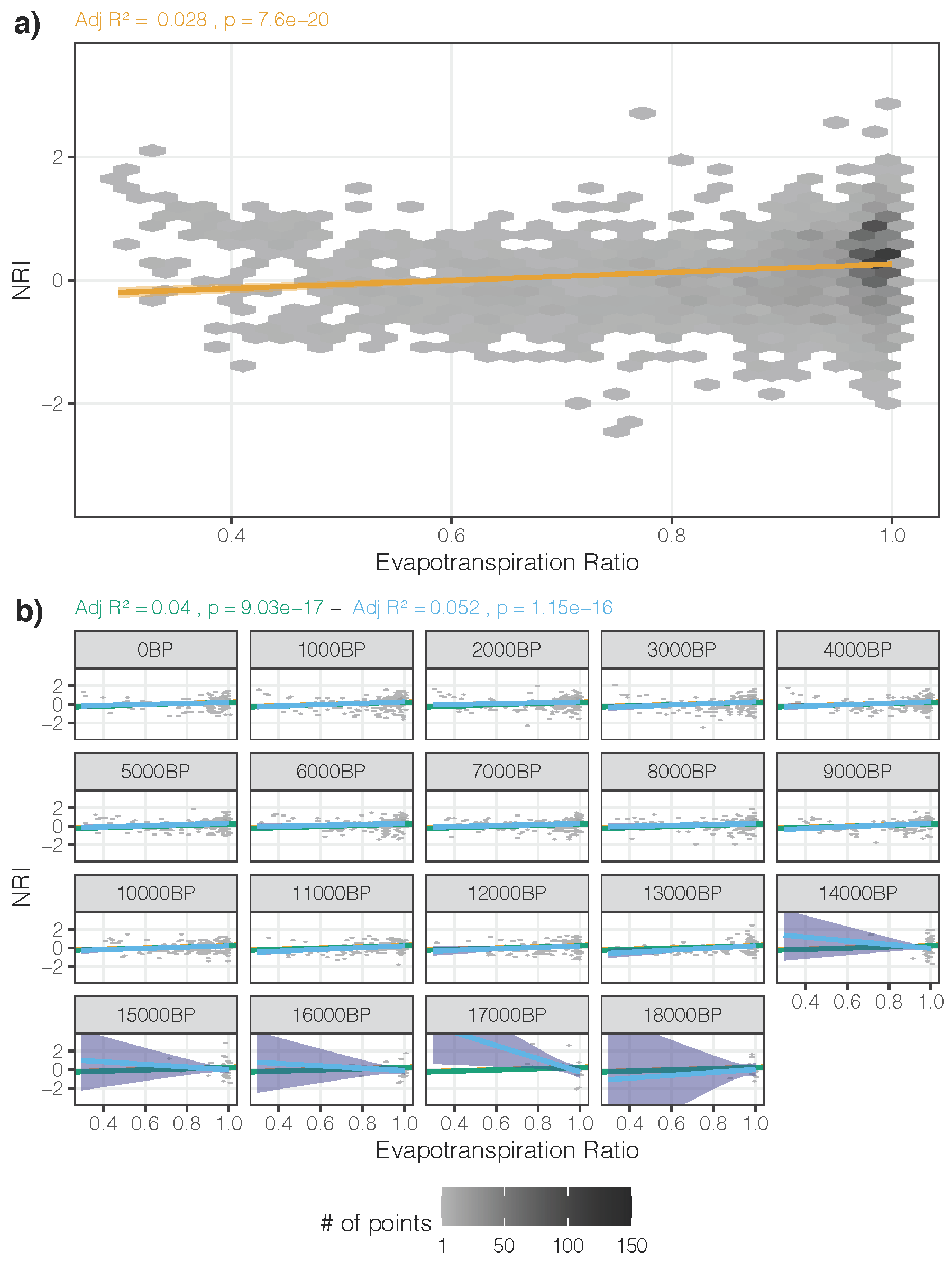

Supplement: S2 Fig — Stable-Relationship (orange line), Stable-Slope (green line) and Changed-Relationship (blue line). Tmin = minimum temperature of the coldest month; Tmax = maximum temperature of the warmest month; Pmin = minimum precipitation of the driest month; Pmax = maximum precipitation of the wettest month; AET = mean yearly actual evapotranspiration; ETR = mean yearly ratio of actual and potential evapotranspiration; WDI = mean yearly water deficit index; DEGLAC = time-since-deglaciation. (ZIP) [file pone.0240957.s002.zip › S2_Figure_Page_6.tiff]

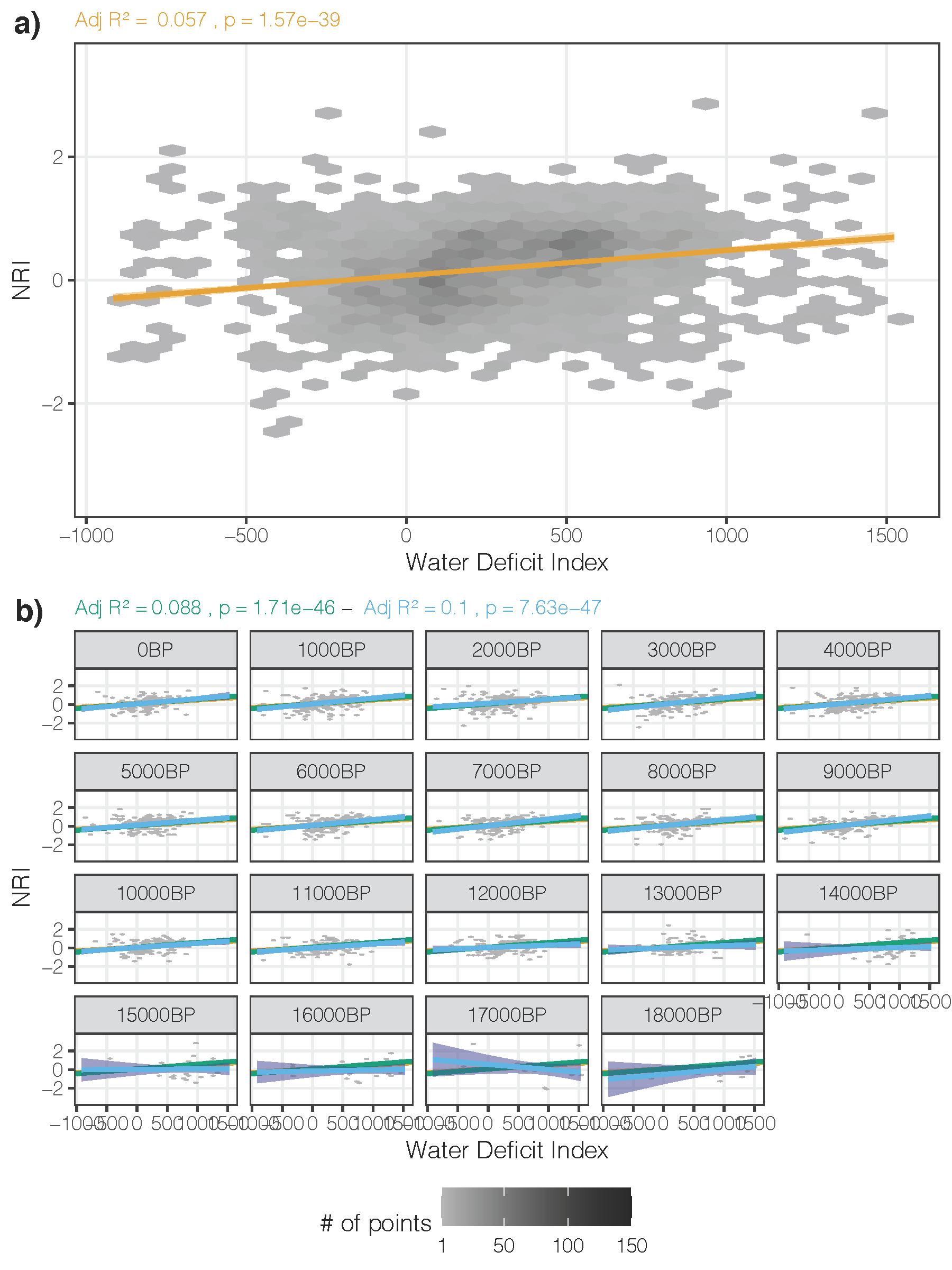

Supplement: S2 Fig — Stable-Relationship (orange line), Stable-Slope (green line) and Changed-Relationship (blue line). Tmin = minimum temperature of the coldest month; Tmax = maximum temperature of the warmest month; Pmin = minimum precipitation of the driest month; Pmax = maximum precipitation of the wettest month; AET = mean yearly actual evapotranspiration; ETR = mean yearly ratio of actual and potential evapotranspiration; WDI = mean yearly water deficit index; DEGLAC = time-since-deglaciation. (ZIP) [file pone.0240957.s002.zip › S2_Figure_Page_7.tiff]

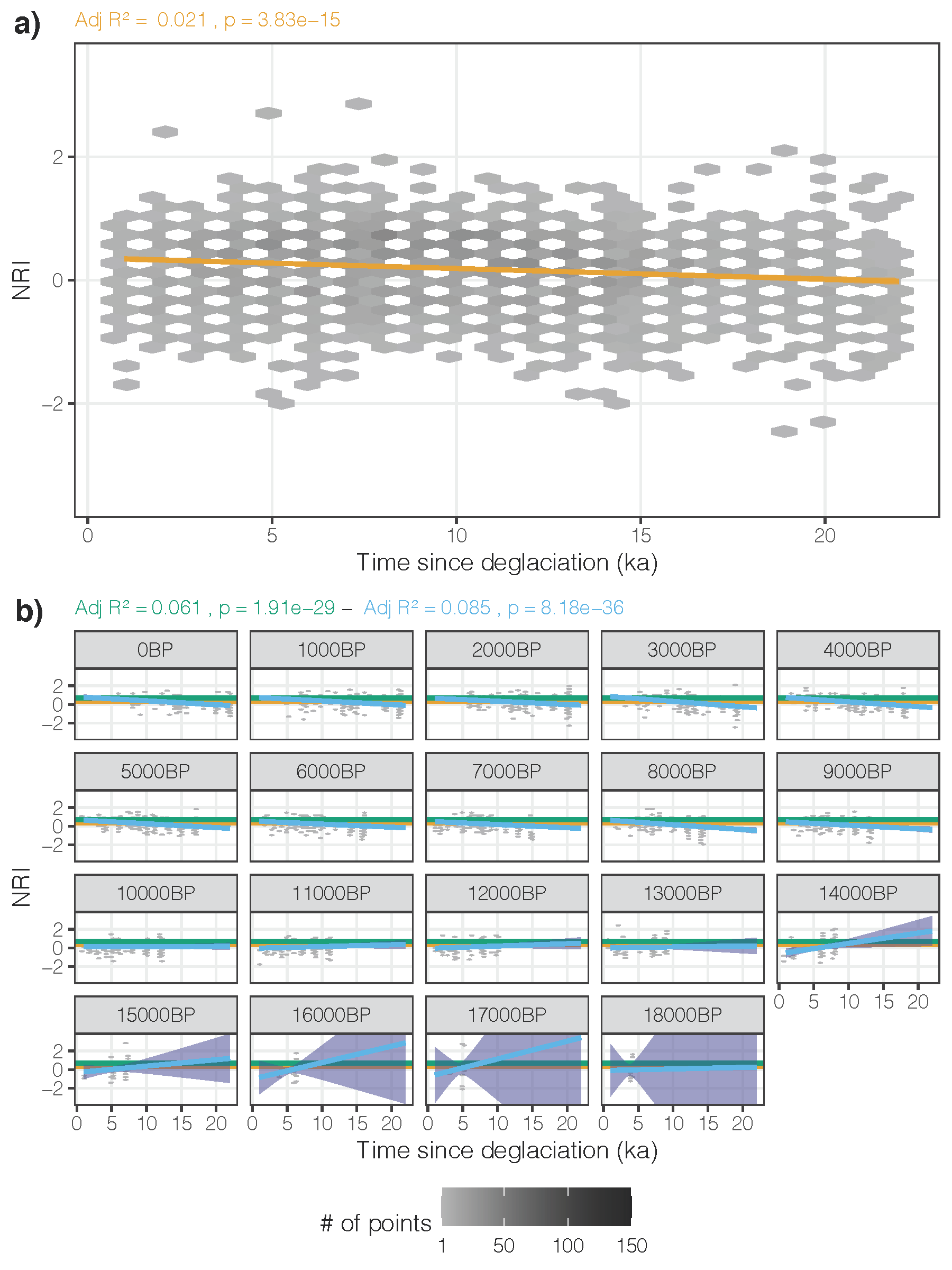

Supplement: S2 Fig — Stable-Relationship (orange line), Stable-Slope (green line) and Changed-Relationship (blue line). Tmin = minimum temperature of the coldest month; Tmax = maximum temperature of the warmest month; Pmin = minimum precipitation of the driest month; Pmax = maximum precipitation of the wettest month; AET = mean yearly actual evapotranspiration; ETR = mean yearly ratio of actual and potential evapotranspiration; WDI = mean yearly water deficit index; DEGLAC = time-since-deglaciation. (ZIP) [file pone.0240957.s002.zip › S2_Figure_Page_8.tiff]

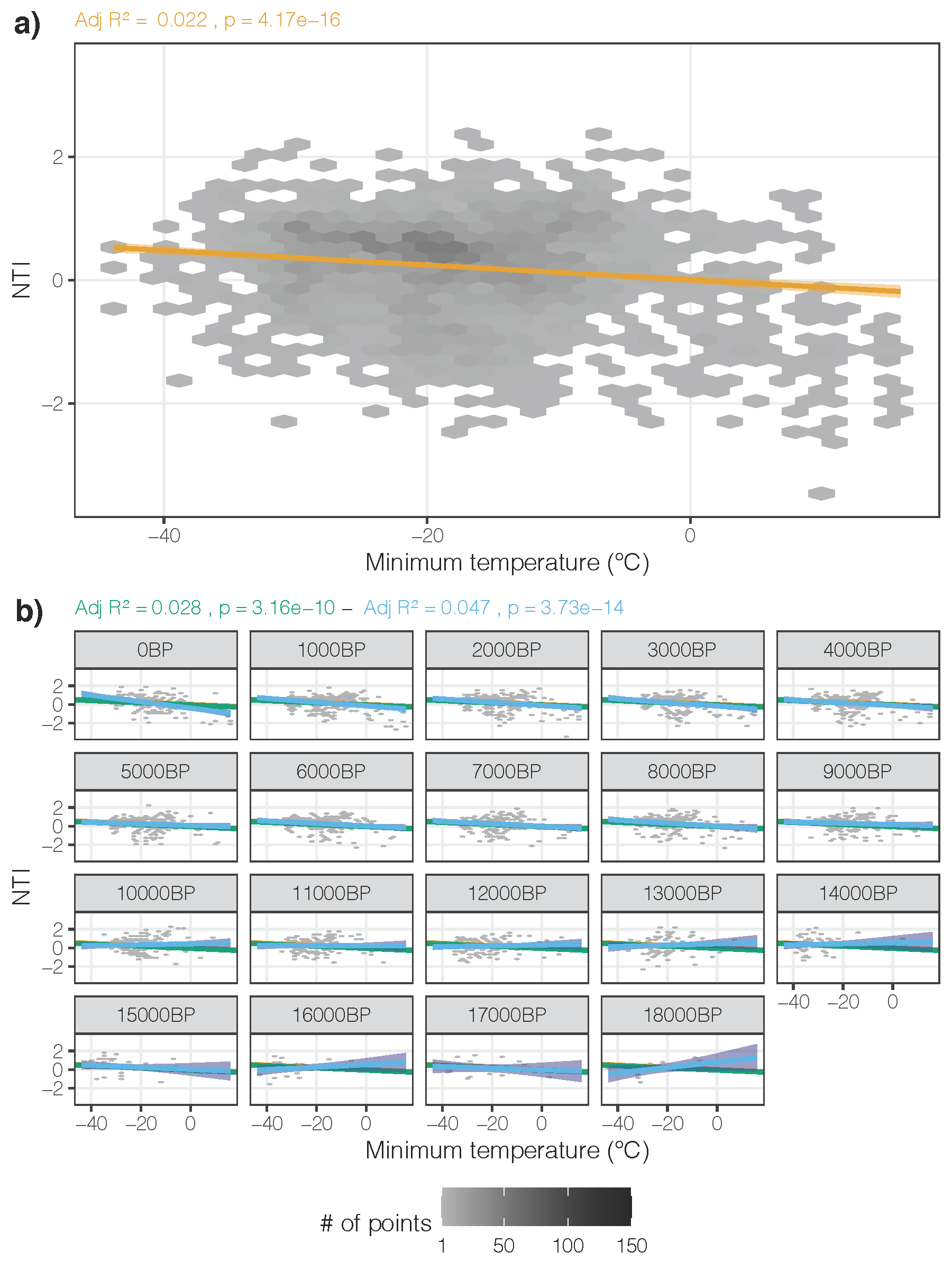

Supplement: S3 Fig — Stable-Relationship (orange line), Stable-Slope (green line), and Changed-Relationship (blue line). Note that each model is fitted to data for all time periods and hence a single adjusted R2 and p-value for each model is presented in the same colors as the lines. Tmin = minimum temperature of the coldest month; Tmax = maximum temperature of the warmest month; Pmin = minimum precipitation of the driest month; Pmax = maximum precipitation of the wettest month; AET = mean yearly actual evapotranspiration; ETR = mean yearly ratio of actual and potential evapotranspiration; WDI = mean yearly water deficit index; DEGLAC = time-since-deglaciation. (ZIP) [file pone.0240957.s003.zip › S3_Figure_Page_1.tiff]

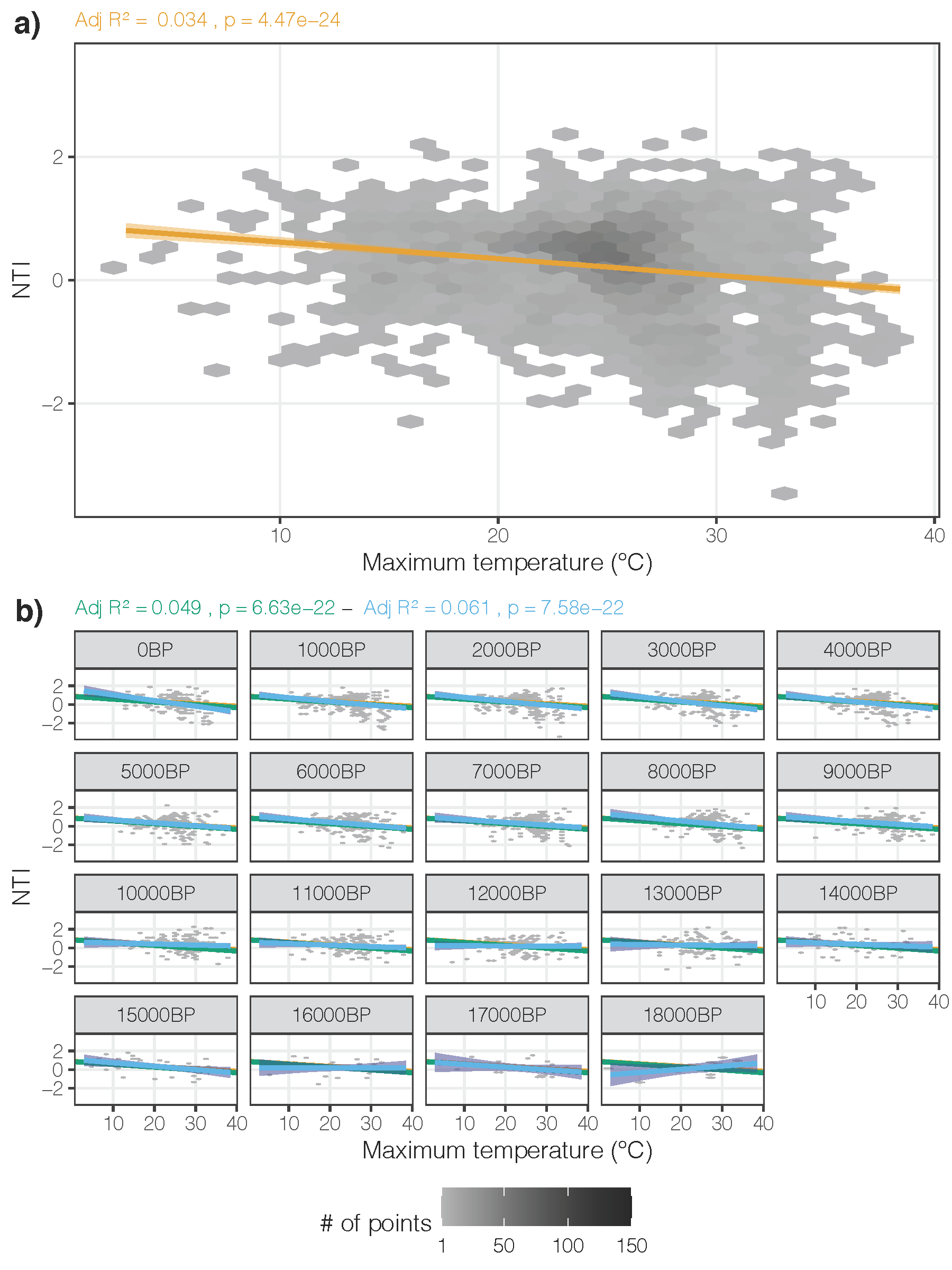

Supplement: S3 Fig — Stable-Relationship (orange line), Stable-Slope (green line), and Changed-Relationship (blue line). Note that each model is fitted to data for all time periods and hence a single adjusted R2 and p-value for each model is presented in the same colors as the lines. Tmin = minimum temperature of the coldest month; Tmax = maximum temperature of the warmest month; Pmin = minimum precipitation of the driest month; Pmax = maximum precipitation of the wettest month; AET = mean yearly actual evapotranspiration; ETR = mean yearly ratio of actual and potential evapotranspiration; WDI = mean yearly water deficit index; DEGLAC = time-since-deglaciation. (ZIP) [file pone.0240957.s003.zip › S3_Figure_Page_2.tiff]

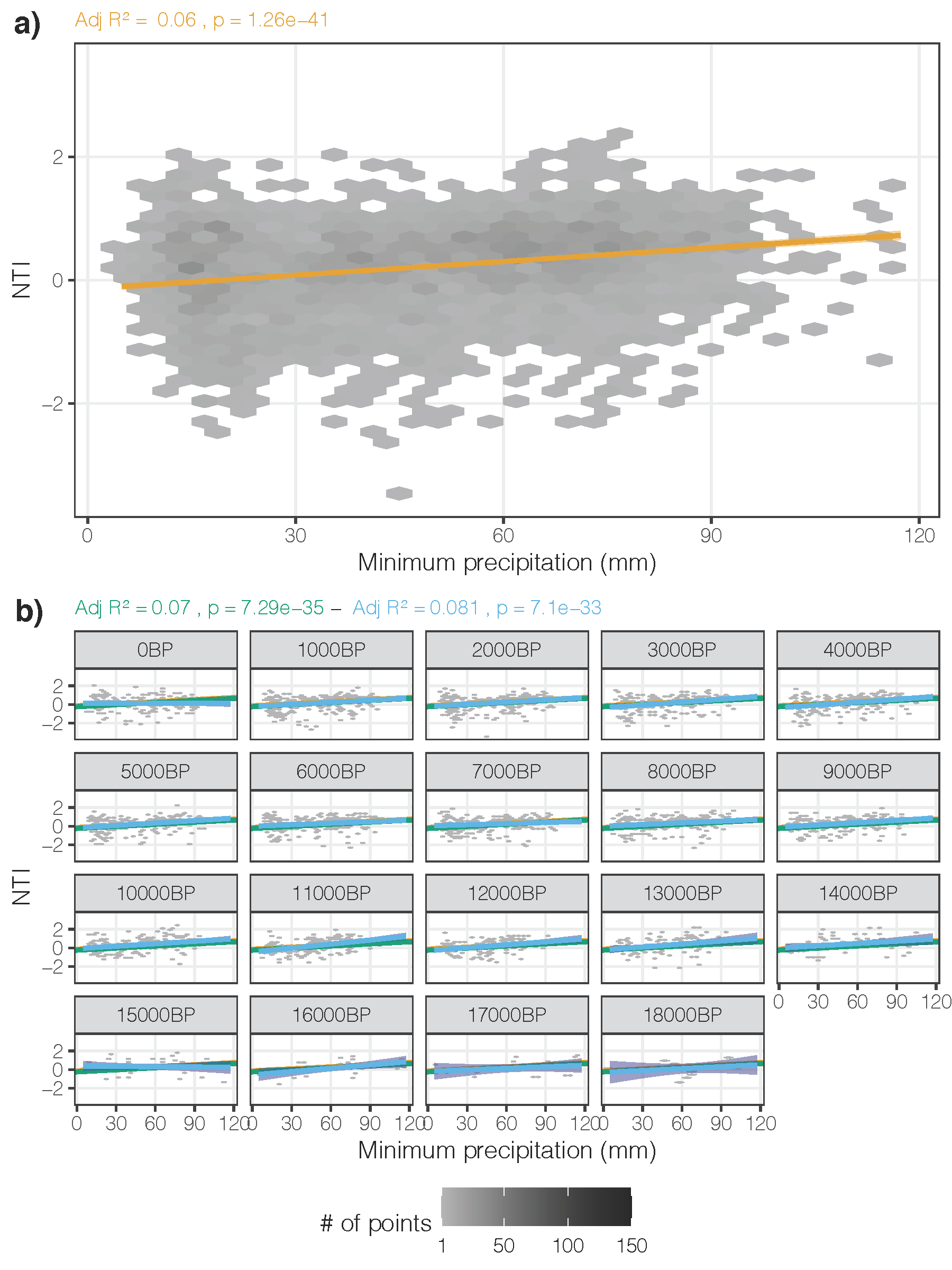

Supplement: S3 Fig — Stable-Relationship (orange line), Stable-Slope (green line), and Changed-Relationship (blue line). Note that each model is fitted to data for all time periods and hence a single adjusted R2 and p-value for each model is presented in the same colors as the lines. Tmin = minimum temperature of the coldest month; Tmax = maximum temperature of the warmest month; Pmin = minimum precipitation of the driest month; Pmax = maximum precipitation of the wettest month; AET = mean yearly actual evapotranspiration; ETR = mean yearly ratio of actual and potential evapotranspiration; WDI = mean yearly water deficit index; DEGLAC = time-since-deglaciation. (ZIP) [file pone.0240957.s003.zip › S3_Figure_Page_3.tiff]

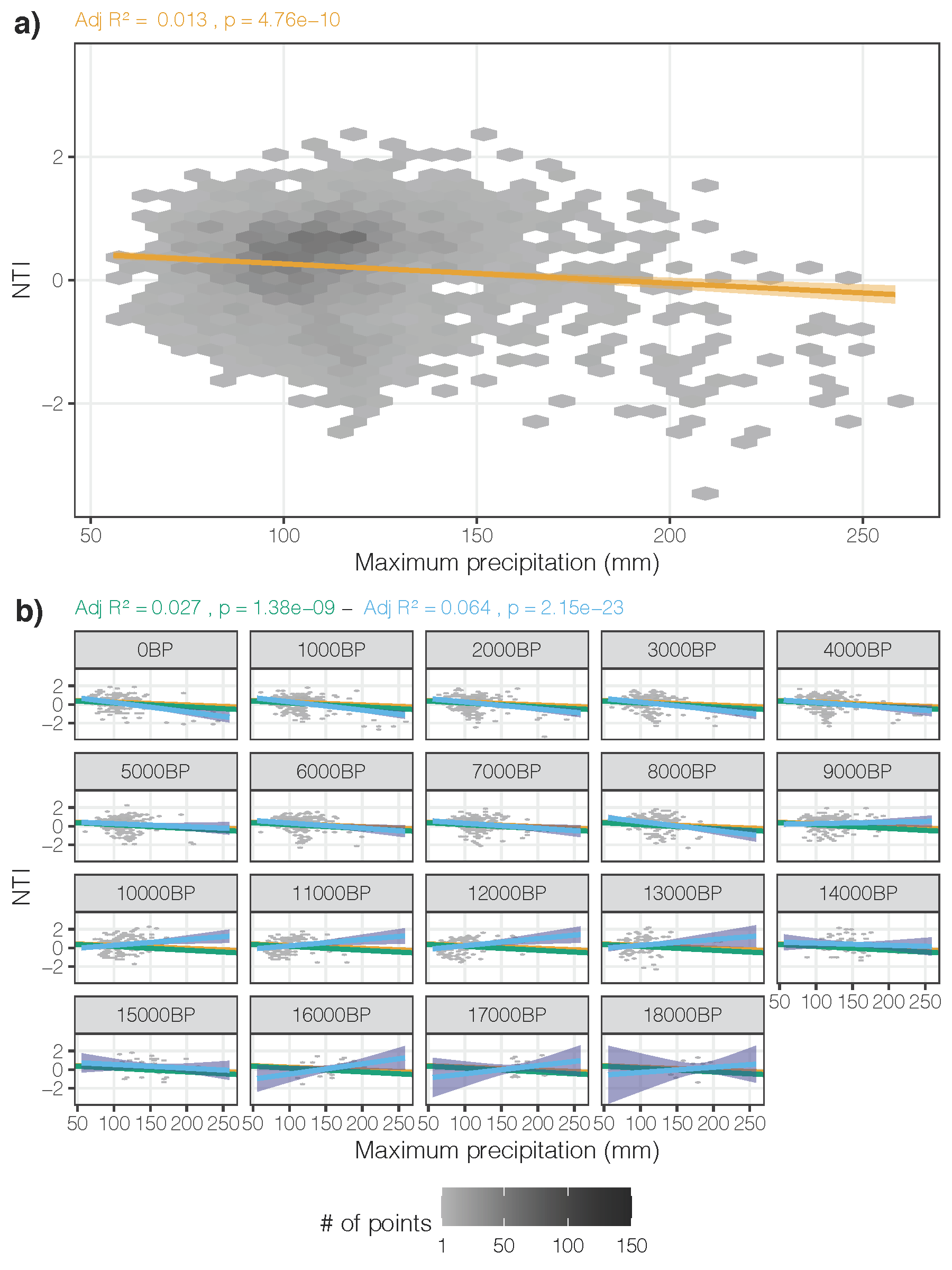

Supplement: S3 Fig — Stable-Relationship (orange line), Stable-Slope (green line), and Changed-Relationship (blue line). Note that each model is fitted to data for all time periods and hence a single adjusted R2 and p-value for each model is presented in the same colors as the lines. Tmin = minimum temperature of the coldest month; Tmax = maximum temperature of the warmest month; Pmin = minimum precipitation of the driest month; Pmax = maximum precipitation of the wettest month; AET = mean yearly actual evapotranspiration; ETR = mean yearly ratio of actual and potential evapotranspiration; WDI = mean yearly water deficit index; DEGLAC = time-since-deglaciation. (ZIP) [file pone.0240957.s003.zip › S3_Figure_Page_4.tiff]

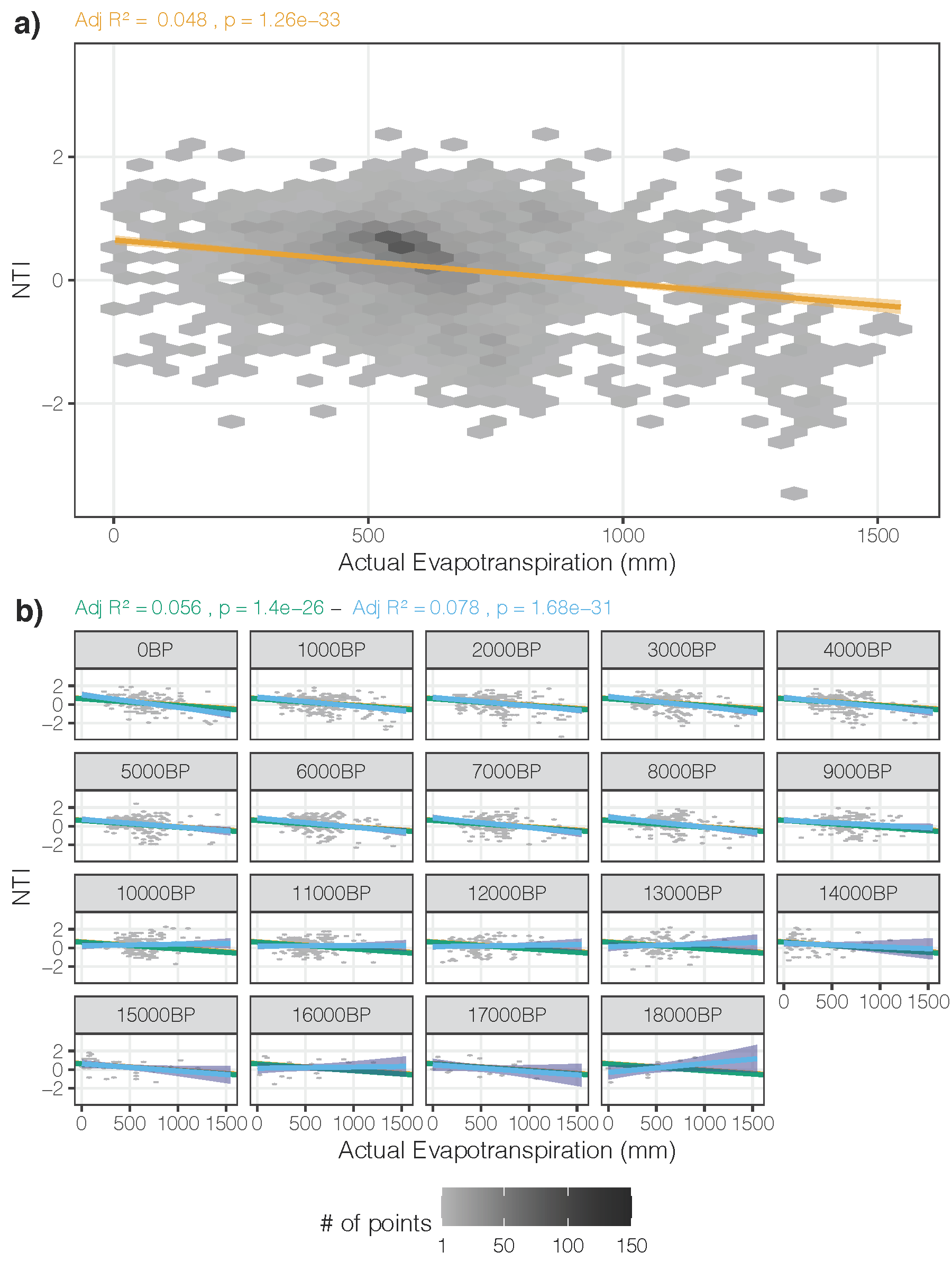

Supplement: S3 Fig — Stable-Relationship (orange line), Stable-Slope (green line), and Changed-Relationship (blue line). Note that each model is fitted to data for all time periods and hence a single adjusted R2 and p-value for each model is presented in the same colors as the lines. Tmin = minimum temperature of the coldest month; Tmax = maximum temperature of the warmest month; Pmin = minimum precipitation of the driest month; Pmax = maximum precipitation of the wettest month; AET = mean yearly actual evapotranspiration; ETR = mean yearly ratio of actual and potential evapotranspiration; WDI = mean yearly water deficit index; DEGLAC = time-since-deglaciation. (ZIP) [file pone.0240957.s003.zip › S3_Figure_Page_5.tiff]

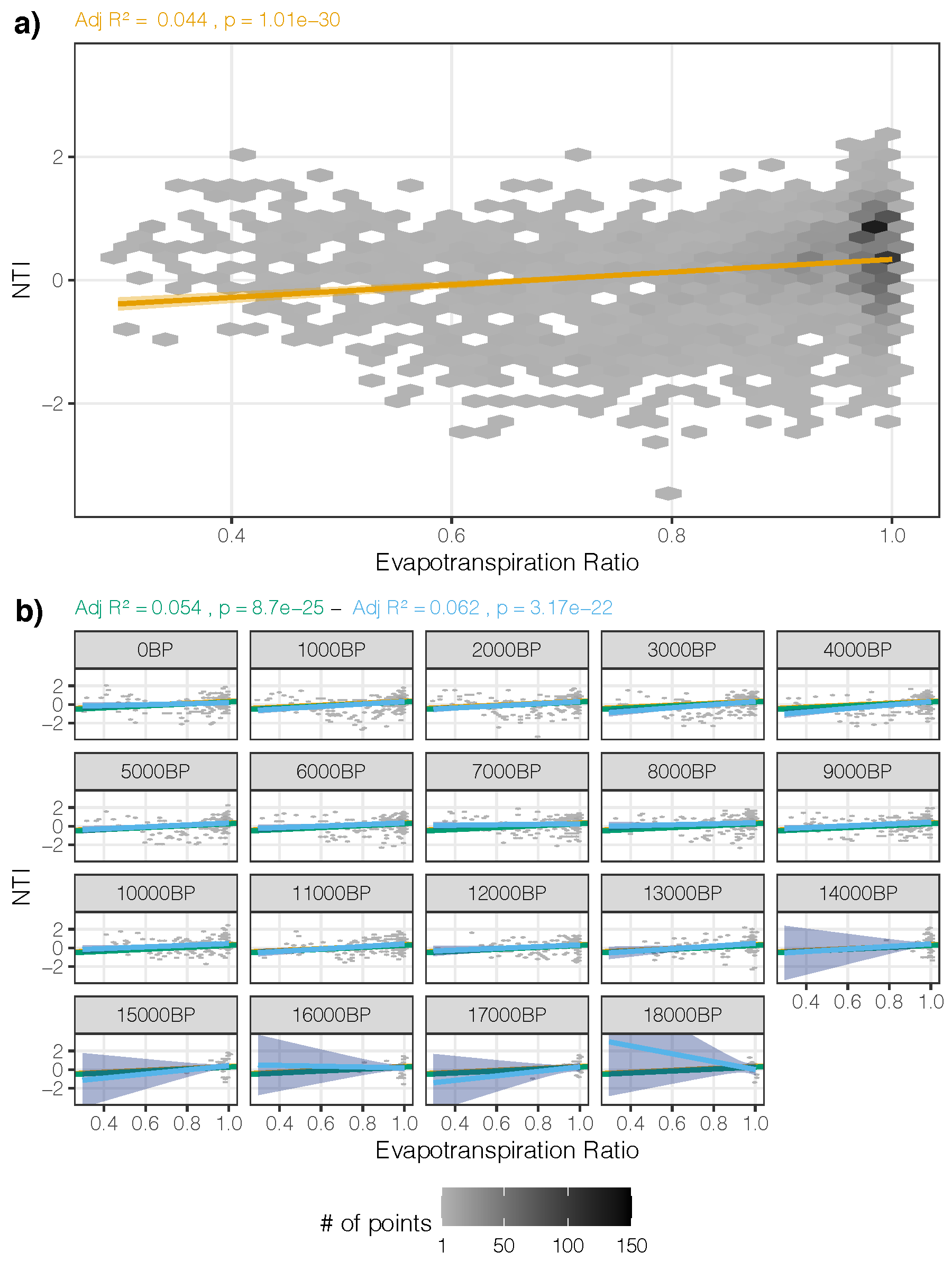

Supplement: S3 Fig — Stable-Relationship (orange line), Stable-Slope (green line), and Changed-Relationship (blue line). Note that each model is fitted to data for all time periods and hence a single adjusted R2 and p-value for each model is presented in the same colors as the lines. Tmin = minimum temperature of the coldest month; Tmax = maximum temperature of the warmest month; Pmin = minimum precipitation of the driest month; Pmax = maximum precipitation of the wettest month; AET = mean yearly actual evapotranspiration; ETR = mean yearly ratio of actual and potential evapotranspiration; WDI = mean yearly water deficit index; DEGLAC = time-since-deglaciation. (ZIP) [file pone.0240957.s003.zip › S3_Figure_Page_6.tiff]

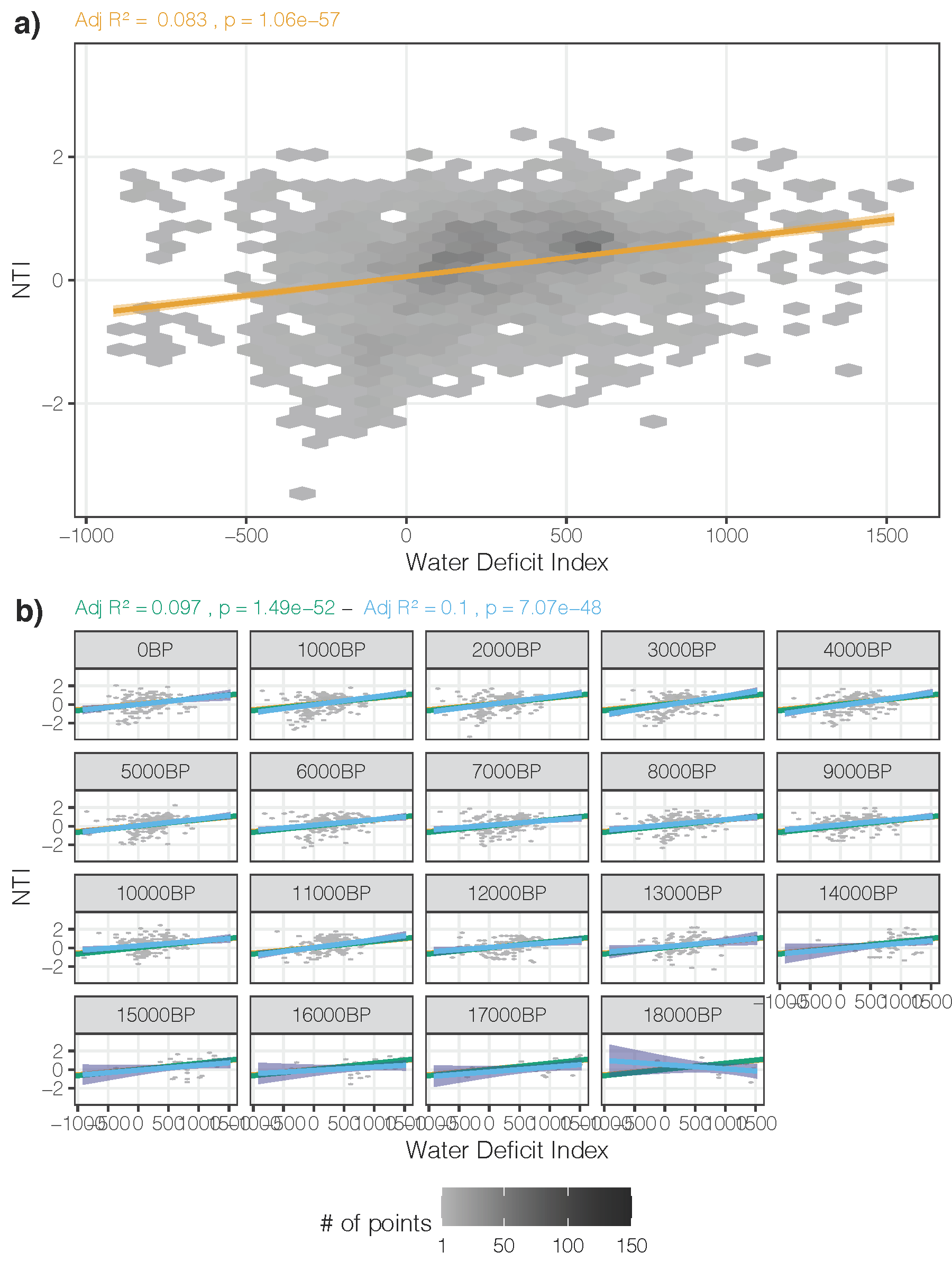

Supplement: S3 Fig — Stable-Relationship (orange line), Stable-Slope (green line), and Changed-Relationship (blue line). Note that each model is fitted to data for all time periods and hence a single adjusted R2 and p-value for each model is presented in the same colors as the lines. Tmin = minimum temperature of the coldest month; Tmax = maximum temperature of the warmest month; Pmin = minimum precipitation of the driest month; Pmax = maximum precipitation of the wettest month; AET = mean yearly actual evapotranspiration; ETR = mean yearly ratio of actual and potential evapotranspiration; WDI = mean yearly water deficit index; DEGLAC = time-since-deglaciation. (ZIP) [file pone.0240957.s003.zip › S3_Figure_Page_7.tiff]

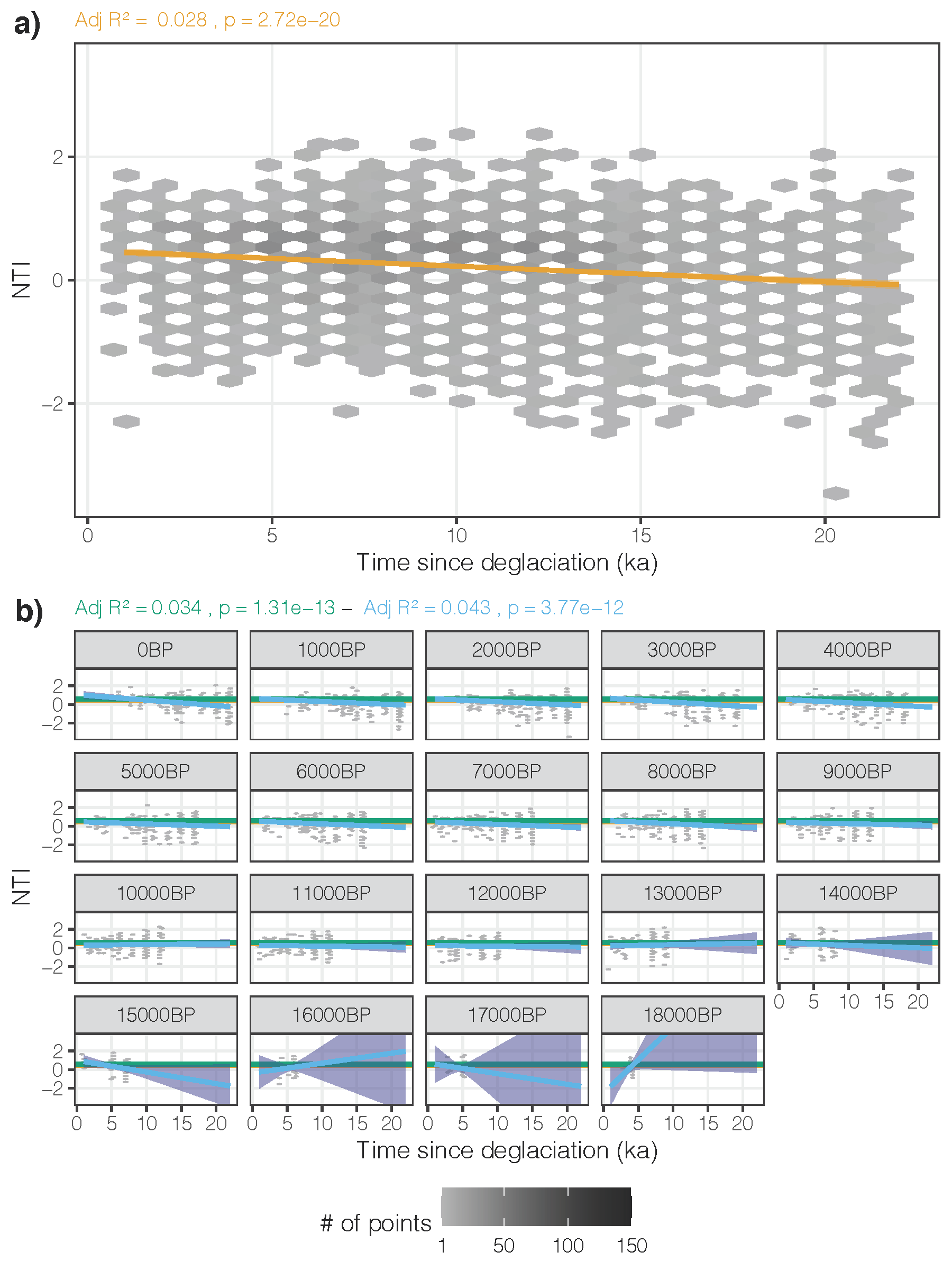

Supplement: S3 Fig — Stable-Relationship (orange line), Stable-Slope (green line), and Changed-Relationship (blue line). Note that each model is fitted to data for all time periods and hence a single adjusted R2 and p-value for each model is presented in the same colors as the lines. Tmin = minimum temperature of the coldest month; Tmax = maximum temperature of the warmest month; Pmin = minimum precipitation of the driest month; Pmax = maximum precipitation of the wettest month; AET = mean yearly actual evapotranspiration; ETR = mean yearly ratio of actual and potential evapotranspiration; WDI = mean yearly water deficit index; DEGLAC = time-since-deglaciation. (ZIP) [file pone.0240957.s003.zip › S3_Figure_Page_8.tiff]

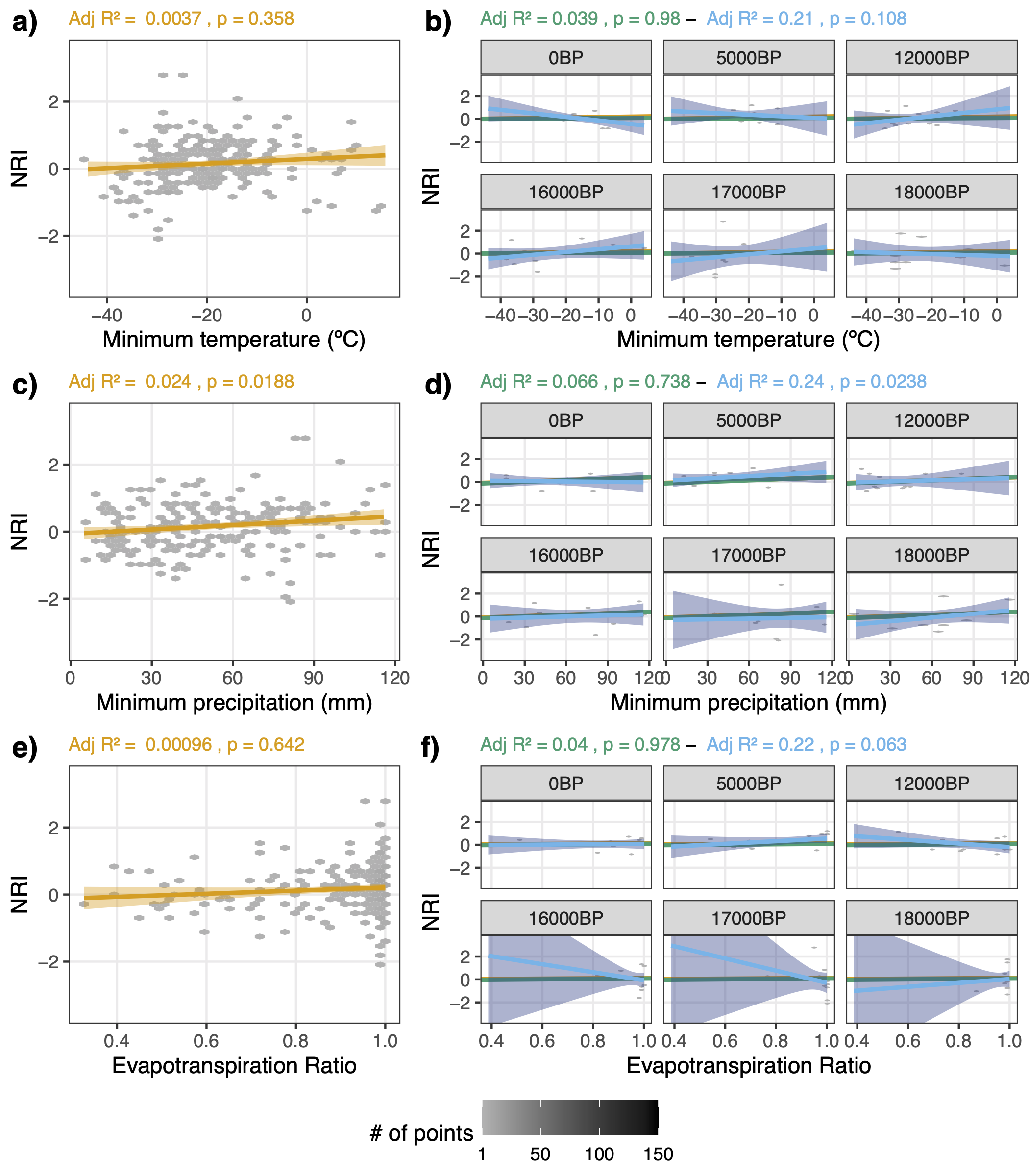

Supplement: S4 Fig — Gray shading in the scatter plots represents the count of points falling in each bin (hexagons). The panels on the left (a, c, e) represent the overall relationship according to ordinary least square regression when pooling all time periods (Stable-Relationship; orange lines). The panels on the right (b, d, f) show the relationship between NRI and climate variables as estimated with the data for different time periods, with green lines representing Stable-Slope Model, blue lines representing Changed-Relationship Model, and the orange line showing the overall relationship from panels on the left for comparison (Stable-Relationship). Shaded areas represent the confidence intervals at 95% for the regression lines. Note that each model is fitted to data for all time periods and hence a single adjusted R2 and p-value for each model is presented in the same colors as the lines. (TIFF) [file pone.0240957.s004.tiff]

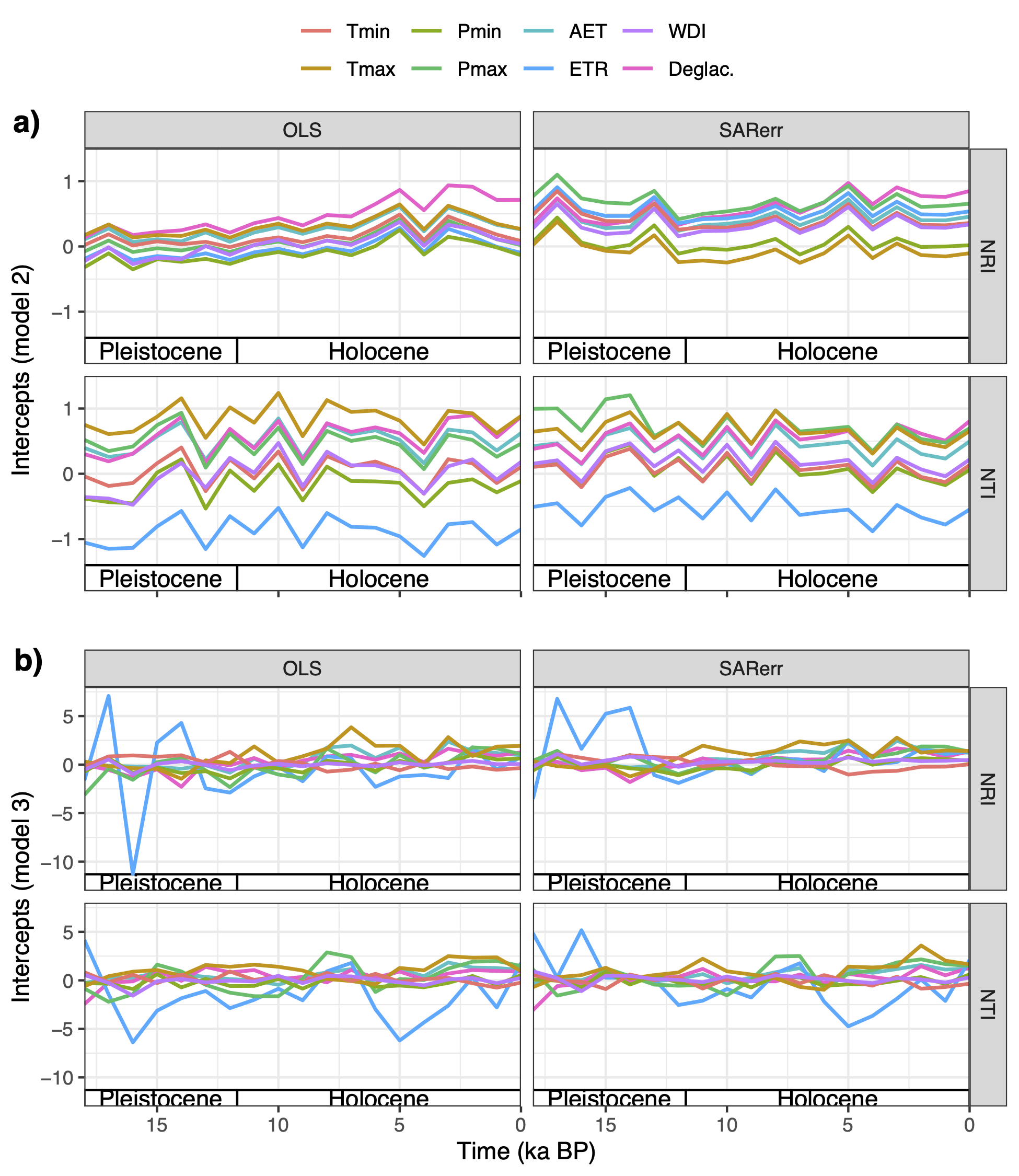

Supplement: S5 Fig — Intercepts of OLS and SAR models for each variable plotted across time for a) Stable-Slope and b) Changed-Relationship models. Tmin = minimum temperature of the coldest month; Tmax = maximum temperature of the warmest month; Pmin = minimum precipitation of the driest month; Pmax = maximum precipitation of the wettest month; AET = mean yearly actual evapotranspiration; ETR = mean yearly ratio of actual and potential evapotranspiration; WDI = mean yearly water deficit index; DEGLAC = time-since-deglaciation. (TIFF) [file pone.0240957.s005.tiff]

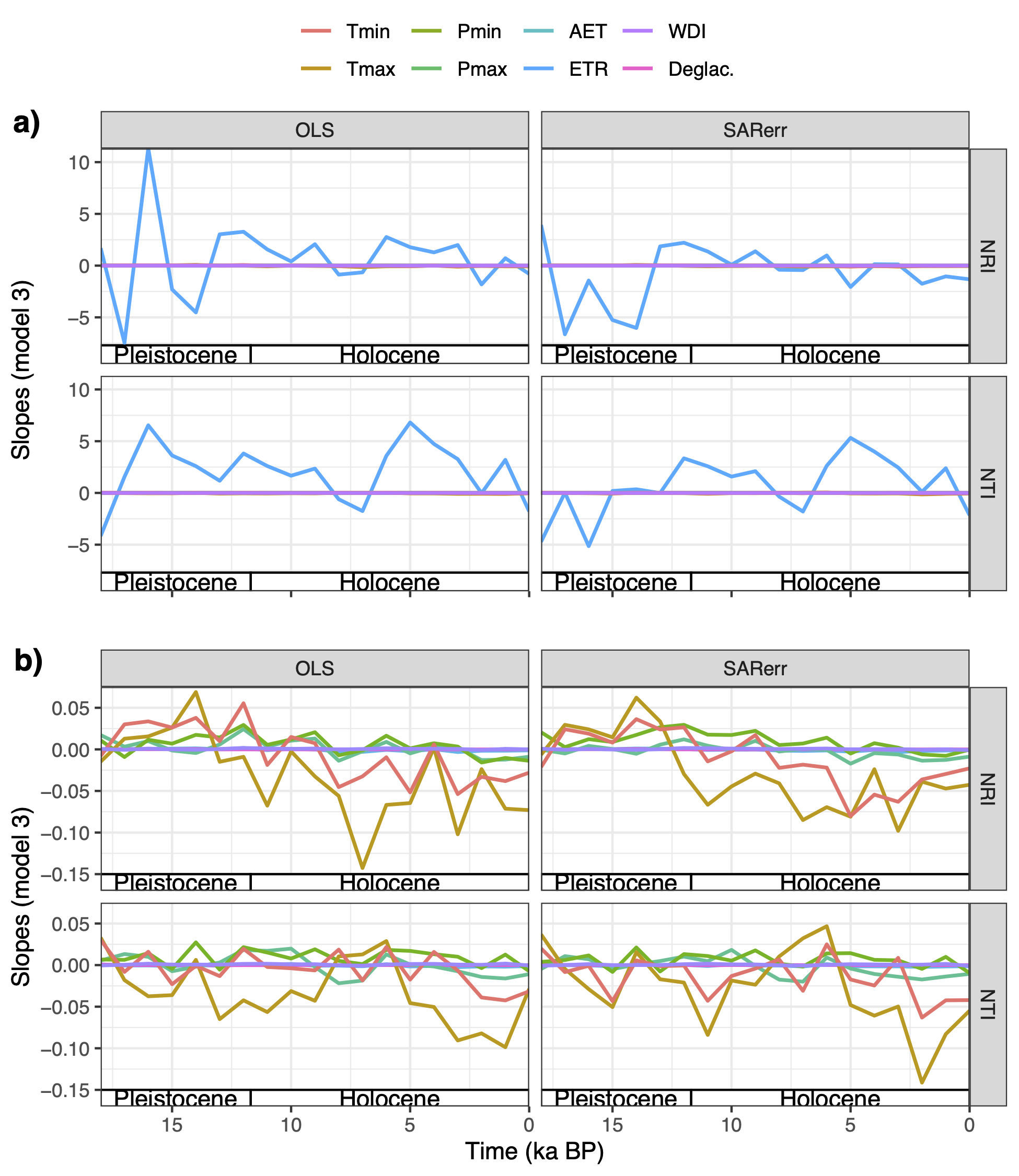

Supplement: S6 Fig — Slopes of OLS and SAR models for each variable plotted across time for the Changed-Relationship models. Note that a) contains slopes for ETR while b) shows the same for all other variables; this was separated for readability as the slope values for ETR varied at a bigger scale than that of other variables. Tmin = minimum temperature of the coldest month; Tmax = maximum temperature of the warmest month; Pmin = minimum precipitation of the driest month; Pmax = maximum precipitation of the wettest month; AET = mean yearly actual evapotranspiration; ETR = mean yearly ratio of actual and potential evapotranspiration; WDI = mean yearly water deficit index; DEGLAC = time-since-deglaciation. (TIFF) [file pone.0240957.s006.tiff]

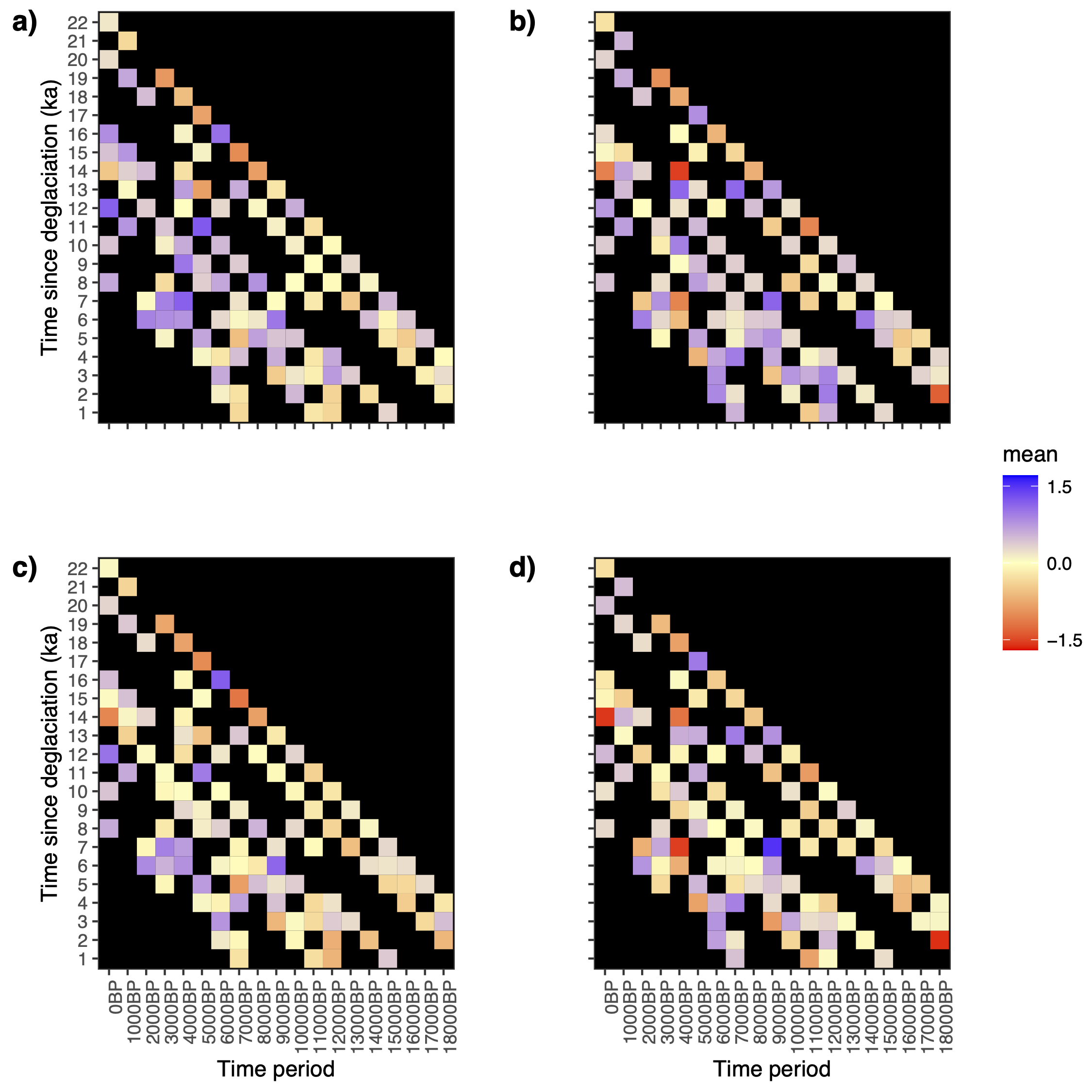

Supplement: S7 Fig — Each cell in the heatmap represents the average a) NRI or b) NTI value for cells of a particular time-since-deglaciation at a particular time period. Black squares indicate absence of the particular time-since-deglaciation class for that time period. The second row of heatmaps represent the average of residuals for c) NRI and d) NTI with the effect of climate variables taken into account using multiple regression models that included all climate variables. Blue colors represent higher PCS values (PCS > 0) and, hence, clustered community structure, whereas red colors represent lower PCS (PCS < 0) values and, hence, overdispersed community structure. (TIFF) [file pone.0240957.s007.tiff]
