# Supplementary material for: Relationships between climate and phylogenetic community structure of fossil pollen assemblages are not constant during the last deglaciation
Source: PLoS One. 2021 Jul 8;16(7):e0240957. doi: 10.1371/journal.pone.0240957 (PMC8266067; doi:10.1371/journal.pone.0240957)
Supplement: S1 Table — (DOCX) [file pone.0240957.s008.docx]

**S1 Table**: **Spatial autocorrelation of raw NRI and NTI values.**

| PCS metric | Distance | Moran’s I |
| --- | --- | --- |
| NRI | 120 | 0.215*** |
|  | 360 | 0.163*** |
|  | 480 | 0.156*** |
| NTI | 120 | 0.287*** |
|  | 360 | 0.243*** |
|  | 480 | 0.214*** |

*** p<0.001; ** p<0.01; * p<0.05; ns non-significant (p>0.05)
