# Supplementary material for: Relationships between climate and phylogenetic community structure of fossil pollen assemblages are not constant during the last deglaciation
Source: PLoS One. 2021 Jul 8;16(7):e0240957. doi: 10.1371/journal.pone.0240957 (PMC8266067; doi:10.1371/journal.pone.0240957)
Supplement: S2 Table — Tmin = minimum temperature of the coldest month; Tmax = maximum temperature of the warmest month; Pmin = minimum precipitation of the driest month; Pmax = maximum precipitation of the wettest month; AET = mean yearly actual evapotranspiration; ETR = mean yearly ratio of actual and potential evapotranspiration; WDI = mean yearly water deficit index; DEGLAC = time-since-deglaciation. (DOCX) [file pone.0240957.s009.docx]

**S2 Table**: **Moran’s I values for residuals of OLS models relating NRI and NTI with all variables (geographic, climatic, and time-since-deglaciation).**

|  |  | NRI | | | NTI | | |
| --- | --- | --- | --- | --- | --- | --- | --- |
| Distance | Var. | Model 1 | Model 2 | Model 3 | Model 1 | Model 2 | Model 3 |
| 120 | Tmin | 0.194*** | 0.182*** | 0.184*** | 0.274*** | 0.274*** | 0.277*** |
|  | Tmax | 0.141*** | 0.131*** | 0.133*** | 0.249*** | 0.245*** | 0.252*** |
|  | Pmin | 0.189*** | 0.174*** | 0.174*** | 0.215*** | 0.215*** | 0.212*** |
|  | Pmax | 0.214*** | 0.204*** | 0.19*** | 0.279*** | 0.28*** | 0.267*** |
|  | AET | 0.165*** | 0.154*** | 0.157*** | 0.25*** | 0.251*** | 0.259*** |
|  | ETR | 0.17*** | 0.159*** | 0.156*** | 0.218*** | 0.216*** | 0.212*** |
|  | WDI | 0.137*** | 0.111*** | 0.107*** | 0.184*** | 0.179*** | 0.182*** |
|  | Deglac. | 0.181*** | 0.156*** | 0.165*** | 0.267*** | 0.267*** | 0.269*** |
| 360 | Tmin | 0.144*** | 0.133*** | 0.148*** | 0.229*** | 0.229*** | 0.232*** |
|  | Tmax | 0.092*** | 0.082*** | 0.094*** | 0.206*** | 0.202*** | 0.21*** |
|  | Pmin | 0.144*** | 0.127*** | 0.131*** | 0.187*** | 0.189*** | 0.187*** |
|  | Pmax | 0.161*** | 0.151*** | 0.152*** | 0.231*** | 0.233*** | 0.217*** |
|  | AET | 0.118*** | 0.109*** | 0.123*** | 0.205*** | 0.206*** | 0.212*** |
|  | ETR | 0.125*** | 0.111*** | 0.112*** | 0.196*** | 0.195*** | 0.192*** |
|  | WDI | 0.096*** | 0.068*** | 0.068*** | 0.155*** | 0.148*** | 0.152*** |
|  | Deglac. | 0.133*** | 0.108*** | 0.121*** | 0.226*** | 0.224*** | 0.228*** |
| 480 | Tmin | 0.141*** | 0.133*** | 0.142*** | 0.201*** | 0.202*** | 0.204*** |
|  | Tmax | 0.091*** | 0.084*** | 0.092*** | 0.179*** | 0.176*** | 0.183*** |
|  | Pmin | 0.138*** | 0.123*** | 0.128*** | 0.165*** | 0.167*** | 0.166*** |
|  | Pmax | 0.154*** | 0.146*** | 0.144*** | 0.201*** | 0.203*** | 0.189*** |
|  | AET | 0.115*** | 0.109*** | 0.116*** | 0.177*** | 0.178*** | 0.182*** |
|  | ETR | 0.125*** | 0.114*** | 0.114*** | 0.174*** | 0.174*** | 0.172*** |
|  | WDI | 0.096*** | 0.074*** | 0.073*** | 0.136*** | 0.13*** | 0.134*** |
|  | Deglac. | 0.129*** | 0.107*** | 0.118*** | 0.196*** | 0.195*** | 0.198*** |

*** p<0.001; ** p<0.01; * p<0.05. ns non-significant (p>0.05)
