# Supplementary material for: Relationships between climate and phylogenetic community structure of fossil pollen assemblages are not constant during the last deglaciation
Source: PLoS One. 2021 Jul 8;16(7):e0240957. doi: 10.1371/journal.pone.0240957 (PMC8266067; doi:10.1371/journal.pone.0240957)
Supplement: S3 Table — Moran’s I values are based on models fitted with error-SAR at the specified distances and are all non-significant (ns). Tmin = minimum temperature of the coldest month; Tmax = maximum temperature of the warmest month; Pmin = minimum precipitation of the driest month; Pmax = maximum precipitation of the wettest month; AET = mean yearly actual evapotranspiration; ETR = mean yearly ratio of actual and potential evapotranspiration; WDI = mean yearly water deficit index; DEGLAC = time-since-deglaciation. (DOCX) [file pone.0240957.s010.docx]

**S3 Table**. **Moran’s I values for residuals of SAR models relating NRI and NTI with seven climate variables**. Moran’s I values are based on models fitted with error-SAR at the specified distances and are all non-significant (ns).

|  |  | NRI | | | NTI | | |
| --- | --- | --- | --- | --- | --- | --- | --- |
| Distance | Var. | Model 1 | Model 2 | Model 3 | Model 1 | Model 2 | Model 3 |
| 120 | Tmin | -0.065ns | -0.064ns | -0.063ns | -0.074ns | -0.075ns | -0.076ns |
|  | Tmax | -0.068ns | -0.066ns | -0.057ns | -0.073ns | -0.073ns | -0.074ns |
|  | Pmin | -0.061ns | -0.061ns | -0.064ns | -0.074ns | -0.073ns | -0.074ns |
|  | Pmax | -0.065ns | -0.065ns | -0.062ns | -0.074ns | -0.075ns | -0.074ns |
|  | AET | -0.063ns | -0.059ns | -0.058ns | -0.072ns | -0.072ns | -0.073ns |
|  | ETR | -0.065ns | -0.064ns | -0.065ns | -0.075ns | -0.076ns | -0.077ns |
|  | WDI | -0.059ns | -0.053ns | -0.052ns | -0.071ns | -0.072ns | -0.072ns |
|  | Deglac. | -0.064ns | -0.058ns | -0.06ns | -0.075ns | -0.075ns | -0.076ns |
| 360 | Tmin | -0.039ns | -0.038ns | -0.033ns | -0.015ns | -0.014ns | -0.012ns |
|  | Tmax | -0.042ns | -0.043ns | -0.034ns | -0.015ns | -0.015ns | -0.013ns |
|  | Pmin | -0.036ns | -0.036ns | -0.036ns | -0.015ns | -0.013ns | -0.013ns |
|  | Pmax | -0.039ns | -0.038ns | -0.033ns | -0.016ns | -0.015ns | -0.014ns |
|  | AET | -0.038ns | -0.036ns | -0.031ns | -0.015ns | -0.015ns | -0.014ns |
|  | ETR | -0.039ns | -0.039ns | -0.038ns | -0.015ns | -0.014ns | -0.014ns |
|  | WDI | -0.036ns | -0.034ns | -0.032ns | -0.014ns | -0.013ns | -0.013ns |
|  | Deglac. | -0.038ns | -0.035ns | -0.035ns | -0.014ns | -0.014ns | -0.013ns |
| 480 | Tmin | -0.011ns | -0.011ns | -0.012ns | -0.001ns | -0.002ns | -0.001ns |
|  | Tmax | -0.013ns | -0.014ns | -0.01ns | -0.003ns | -0.002ns | -0.001ns |
|  | Pmin | -0.01ns | -0.01ns | -0.009ns | -0.003ns | -0.003ns | -0.003ns |
|  | Pmax | -0.01ns | -0.01ns | -0.01ns | -0.003ns | -0.003ns | -0.003ns |
|  | AET | -0.011ns | -0.01ns | -0.01ns | -0.002ns | -0.002ns | -0.001ns |
|  | ETR | -0.01ns | -0.01ns | -0.01ns | -0.002ns | -0.001ns | -0.001ns |
|  | WDI | -0.009ns | -0.008ns | -0.007ns | -0.001ns | -0.001ns | 0ns |
|  | Deglac. | -0.01ns | -0.01ns | -0.011ns | -0.002ns | -0.002ns | -0.002ns |

*** p<0.001; ** p<0.01; * p<0.05. ns = non-significant (p>0.05)
