# Supplementary material for: Relationships between climate and phylogenetic community structure of fossil pollen assemblages are not constant during the last deglaciation
Source: PLoS One. 2021 Jul 8;16(7):e0240957. doi: 10.1371/journal.pone.0240957 (PMC8266067; doi:10.1371/journal.pone.0240957)
Supplement: S4 Table — AIC values of SAR are based on models fitted with error-SAR at the specified distances. The lowest AIC values for each climatic variable and PCS metric (i.e., NRI and NTI) is highlighted in bold. Tmin = minimum temperature of the coldest month; Tmax = maximum temperature of the warmest month; Pmin = minimum precipitation of the driest month; Pmax = maximum precipitation of the wettest month; AET = mean yearly actual evapotranspiration; ETR = mean yearly ratio of actual and potential evapotranspiration; WDI = mean yearly water deficit index; DEGLAC = time-since-deglaciation. (DOCX) [file pone.0240957.s011.docx]

**S4 Table**. **AIC values for OLS and SAR models relating NRI and NTI with seven climate variables.** AIC values of SAR are based on models fitted with error-SAR at the specified distances. The lowest AIC values for each climatic variable and PCS metric (i.e., NRI and NTI) is highlighted in bold.

|  |  | NRI | | | | NTI | | | |
| --- | --- | --- | --- | --- | --- | --- | --- | --- | --- |
|  |  | OLS | SAR  120km | SAR  360 km | SAR  480 km | OLS | SAR  120 km | SAR  360 km | SAR  480 km |
| Model 1 | Tmin | 5347.5 | 4943.5 | 4976.9 | **4926.5** | 6614.7 | 5955.4 | **5921.9** | 5963 |
|  | Tmax | 5259.1 | 4941.8 | 4965.8 | **4916** | 6578.4 | 5971.9 | **5941.4** | 5978.5 |
|  | Pmin | 5305.2 | 4935.9 | 4956 | **4904.7** | 6498 | 5976.9 | **5938.9** | 5965.4 |
|  | Pmax | 5381.2 | 4943.7 | 4973.3 | **4923.8** | 6642.1 | 5965.7 | **5940.4** | 5977.3 |
|  | AET | 5284.3 | 4942.7 | 4976.9 | **4926.2** | 6534.7 | 5945.4 | **5913.9** | 5954.3 |
|  | ETR | 5298.9 | 4943.8 | 4977.4 | **4924.1** | 6548 | 5976.4 | **5940.3** | 5970.8 |
|  | WDI | 5208.9 | 4936.8 | 4966.9 | **4914.6** | 6424.3 | 5968.7 | **5927.7** | 5962.4 |
|  | Deglac. | 5320.3 | 4942.5 | 4973.9 | **4922.8** | 6595.6 | 5950.9 | **5901.7** | 5944.6 |
| Model 2 | Tmin | 5328.8 | 4963.9 | 4996.2 | **4943.6** | 6632.1 | 5971.8 | **5931.9** | 5969.2 |
|  | Tmax | 5237.6 | 4964.3 | 4979.4 | **4931.7** | 6568.8 | 5977.6 | **5932.8** | 5965.2 |
|  | Pmin | 5302.1 | 4958.4 | 4978.9 | **4928.4** | 6502.7 | 5983 | **5920.1** | 5940.6 |
|  | Pmax | 5383.2 | 4964.5 | 4995.2 | **4945.1** | 6635.8 | 5960.3 | **5921.9** | 5961.7 |
|  | AET | 5249.2 | 4956.3 | 4992.5 | **4940.1** | 6544.6 | 5956.7 | **5923.3** | 5963.4 |
|  | ETR | 5297.2 | 4964.5 | 4995.9 | **4944.2** | 6553.8 | 5982.8 | **5931.4** | 5964.4 |
|  | WDI | 5146.1 | 4946.9 | 4982.3 | **4926.5** | 6415.1 | 5979.7 | **5930** | 5966.8 |
|  | Deglac. | 5231.3 | 4947.4 | 4978.7 | **4923.6** | 6613.1 | 5972.2 | **5923** | 5965.3 |
| Model 3 | Tmin | 5173.6 | 4818.5 | 4781.3 | **4743.3** | 6610.3 | 5941.2 | **5886.2** | 5932.6 |
|  | Tmax | 5130.8 | 4892.5 | 4885.8 | **4847** | 6565 | 5962.6 | **5908** | 5947.7 |
|  | Pmin | 5260.6 | 4910.1 | 4913.7 | **4861.3** | 6504.1 | 5987.3 | **5924.9** | 5943.7 |
|  | Pmax | 5300.1 | 4910.1 | 4890 | **4861.8** | 6556.3 | 5914.1 | **5881.8** | 5923.8 |
|  | AET | 5119.3 | 4834.2 | 4799 | **4774** | 6511.5 | 5895.3 | **5848.3** | 5899.1 |
|  | ETR | 5296.4 | 4966.5 | 4986.9 | **4937** | 6562.9 | 5997.8 | **5936.1** | 5956.4 |
|  | WDI | 5131.5 | 4945.7 | 4974.7 | **4920.7** | 6424.9 | 5983 | **5922.1** | 5959.7 |
|  | Deglac. | 5189.6 | 4881.7 | 4878.8 | **4832.9** | 6622.7 | 5972.4 | **5912.6** | 5954.8 |
