# Supplementary material for: Relationships between climate and phylogenetic community structure of fossil pollen assemblages are not constant during the last deglaciation
Source: PLoS One. 2021 Jul 8;16(7):e0240957. doi: 10.1371/journal.pone.0240957 (PMC8266067; doi:10.1371/journal.pone.0240957)
Supplement: S5 Table — Each combination of PCS metric and climate variable was modeled three times, allowing distinct levels of variation to study the evolution of the parameters through time: stable-relationship, stable-slope, and changed relationship. SAR models reported here were fit selecting neighbors at distances of 480 km for NRI and NTI. Tmin = minimum temperature of the coldest month; Tmax = maximum temperature of the warmest month; Pmin = minimum precipitation of the driest month; Pmax = maximum precipitation of the wettest month; AET = mean yearly actual evapotranspiration; ETR = mean yearly ratio of actual and potential evapotranspiration; WDI = mean yearly water deficit index; DEGLAC = time-since-deglaciation. (DOCX) [file pone.0240957.s012.docx]

**S5 Table**. **Parameters of ordinary least square regression (OLS) and spatial autoregressive (SAR) models relating two metrics of phylogenetic community structure (PCS; net relatedness index – NRI – and nearest taxon index – NTI) with seven climate variables and equal sample size through time (n=12 in each time period).** Each combination of PCS metric and climate variable was modeled three times, allowing distinct levels of variation to study the evolution of the parameters through time: stable-relationship, stable-slope, and changed relationship. SAR models reported here were fit selecting neighbors at distances of 480 km for NRI and NTI.

| PCS  metric | Model  type | Var. | Stable-Relationship | | | | Stable-Slope | | | Changed-  Relationship | |
| --- | --- | --- | --- | --- | --- | --- | --- | --- | --- | --- | --- |
|  |  |  | Int. | Slope | Adj. R^2^ | P val. | Slope | Adj. R^2^ | P val. | Adj. R^2^ | P val. |
|  |  | Tmin | 0,198 | 0,004 | 0,004 | ns | 0,002 | 0,039 | ns | 0,207 | ns |
|  |  | Tmax | 0,256 | -0,006 | 0,003 | ns | -0,008 | 0,043 | ns | 0,172 | ns |
|  |  | Pmin | -0,082 | 0,004 | 0,024 | * | 0,005 | 0,066 | ns | 0,237 | * |
|  |  | Pmax | 0,184 | -0,001 | 0,001 | ns | 0 | 0,038 | ns | 0,177 | ns |
|  |  | AET | 0,19 | 0 | 0,003 | ns | 0 | 0,047 | ns | 0,213 | ns |
|  |  | ETR | -0,014 | 0,146 | 0,001 | ns | 0,178 | 0,04 | ns | 0,218 | ns |
|  |  | WDI | 0,079 | 0 | 0,004 | ns | 0 | 0,054 | ns | 0,18 | ns |
|  |  | Deglac. | 0,193 | 0 | 0,004 | ns | 0 | 0,075 | ns | 0,172 | ns |
|  | SARerr  (480 km) | Tmin | 0,34 | 0,002 | 0,178 | ns | 0,006 | 0,22 | ns | 0,354 | ns |
|  |  | Tmax | 0,188 | 0,005 | 0,179 | ns | 0,014 | 0,224 | ns | 0,301 | ns |
|  |  | Pmin | 0,063 | 0,005 | 0,19 | ns | 0,006 | 0,23 | * | 0,362 | ns |
|  |  | Pmax | 0,448 | -0,001 | 0,179 | ns | -0,003 | 0,223 | ns | 0,311 | ns |
|  |  | AET | 0,379 | 0 | 0,179 | ns | 0 | 0,218 | ns | 0,333 | ns |
|  |  | ETR | 0,485 | -0,201 | 0,179 | ns | -0,26 | 0,219 | ns | 0,363 | ns |
|  |  | WDI | 0,31 | 0 | 0,179 | ns | 0 | 0,218 | ns | 0,337 | ns |
|  |  | Deglac. | 0,374 | 0 | 0,18 | ns | 0 | 0,226 | ns | 0,295 | ns |
|  | OLS | Tmin | 0,041 | -0,008 | 0,013 | ns | -0,008 | 0,082 | ns | 0,151 | ns |
|  |  | Tmax | 0,774 | -0,023 | 0,033 | ** | -0,026 | 0,106 | ns | 0,17 | ns |
|  |  | Pmin | -0,144 | 0,007 | 0,064 | *** | 0,008 | 0,141 | * | 0,238 | * |
|  |  | Pmax | 0,324 | -0,001 | 0,002 | ns | -0,002 | 0,075 | ns | 0,219 | ns |
|  |  | AET | 0,502 | -0,001 | 0,031 | ** | -0,001 | 0,098 | ns | 0,18 | ns |
|  |  | ETR | -0,796 | 1,134 | 0,048 | *** | 1,246 | 0,12 | ns | 0,261 | ** |
|  |  | WDI | 0,064 | 0 | 0,059 | *** | 0,001 | 0,148 | * | 0,246 | * |
|  |  | Deglac. | 0,398 | 0 | 0,016 | ns | 0 | 0,099 | ns | 0,17 | ns |
|  | SARerr  (480 km) | Tmin | 0,124 | -0,006 | 0,131 | ns | -0,006 | 0,19 | ns | 0,272 | ** |
|  |  | Tmax | 0,507 | -0,011 | 0,132 | ns | -0,015 | 0,193 | ns | 0,274 | ns |
|  |  | Pmin | -0,017 | 0,005 | 0,138 | ns | 0,005 | 0,199 | * | 0,279 | ns |
|  |  | Pmax | 0,356 | -0,001 | 0,128 | ns | -0,003 | 0,194 | ns | 0,3 | ns |
|  |  | AET | 0,382 | 0 | 0,133 | ns | 0 | 0,191 | ns | 0,274 | * |
|  |  | ETR | -0,598 | 0,945 | 0,144 | * | 0,953 | 0,201 | * | 0,287 | ns |
|  |  | WDI | 0,173 | 0 | 0,134 | ns | 0 | 0,194 | ns | 0,269 | ns |
|  |  | Deglac. | 0,346 | 0 | 0,134 | ns | 0 | 0,195 | ns | 0,269 | ns |
