# Supplementary material for: Relationships between climate and phylogenetic community structure of fossil pollen assemblages are not constant during the last deglaciation
Source: PLoS One. 2021 Jul 8;16(7):e0240957. doi: 10.1371/journal.pone.0240957 (PMC8266067; doi:10.1371/journal.pone.0240957)
Supplement: S6 Table — Each ANOVA was run for each combination of PCS metric and climate variable comparing three different models that allow distinct levels of variation to study the evolution of the regression parameters through time: stable-relationship, stable-slope, and changed relationship. SAR models reported here were fit selecting neighbors at distances of 360 km. Tmin = minimum temperature of the coldest month; Tmax = maximum temperature of the warmest month; Pmin = minimum precipitation of the driest month; Pmax = maximum precipitation of the wettest month; AET = mean yearly actual evapotranspiration; ETR = mean yearly ratio of actual and potential evapotranspiration; WDI = mean yearly water deficit index; DEGLAC = time-since-deglaciation. (DOCX) [file pone.0240957.s013.docx]

**S6 Table**. **ANOVA-based model selection of ordinary least square regression (OLS) and spatial autoregressive (SAR) models relating net relatedness index (NRI) and nearest taxon index (NTI) with seven climate variables and equal sample size through time (n=12 in each time period).** Each ANOVA was run for each combination of PCS metric and climate variable comparing three different models that allow distinct levels of variation to study the evolution of the regression parameters through time: stable-relationship, stable-slope, and changed relationship. SAR models reported here were fit selecting neighbors at distances of 360 km.

| PCS  metric | Model  Type | Var. | Stable-Relationship | | Stable-Slope | | Changed-Relationship | | Selected  Model |
| --- | --- | --- | --- | --- | --- | --- | --- | --- | --- |
|  |  |  | Res df  (OLS)  Df  (SAR) | F  (OLS)  L ratio  (SAR) | Res df  (OLS)  Df  (SAR) | F  (OLS)  L ratio  (SAR) | Res df  (OLS)  Df  (SAR) | F  (OLS)  L ratio  (SAR) |  |
| NRI | OLS | Tmin | 226 | NA | 208 | 0,468 | 190 | 2,234 | 3** |
|  |  | Tmax | 226 | NA | 208 | 0,511 | 190 | 1,645 | 1 |
|  |  | Pmin | 226 | NA | 208 | 0,577 | 190 | 2,364 | 3** |
|  |  | Pmax | 226 | NA | 208 | 0,484 | 190 | 1,78 | 3* |
|  |  | AET | 226 | NA | 208 | 0,597 | 190 | 2,234 | 3** |
|  |  | ETR | 226 | NA | 208 | 0,521 | 190 | 2,415 | 3** |
|  |  | WDI | 226 | NA | 208 | 0,639 | 190 | 1,623 | 1 |
|  |  | Deglac. | 226 | NA | 208 | 0,905 | 190 | 1,238 | 1 |
|  | SARerr  (480 km) | Tmin | 4 | NA | 22 | 11,84 | 40 | 42,99 | 3*** |
|  |  | Tmax | 4 | NA | 22 | 12,71 | 40 | 23,916 | 1 |
|  |  | Pmin | 4 | NA | 22 | 11,731 | 40 | 42,869 | 3*** |
|  |  | Pmax | 4 | NA | 22 | 12,58 | 40 | 27,342 | 1 |
|  |  | AET | 4 | NA | 22 | 11,243 | 40 | 36,207 | 3** |
|  |  | ETR | 4 | NA | 22 | 11,374 | 40 | 46,674 | 3*** |
|  |  | WDI | 4 | NA | 22 | 11,195 | 40 | 37,525 | 3** |
|  |  | Deglac. | 4 | NA | 22 | 13,077 | 40 | 21,254 | 1 |
| NTI | OLS | Tmin | 226 | NA | 208 | 0,848 | 190 | 0,865 | 1 |
|  |  | Tmax | 226 | NA | 208 | 0,918 | 190 | 0,823 | 1 |
|  |  | Pmin | 226 | NA | 208 | 1,063 | 190 | 1,345 | 1 |
|  |  | Pmax | 226 | NA | 208 | 0,995 | 190 | 1,945 | 3* |
|  |  | AET | 226 | NA | 208 | 0,851 | 190 | 1,054 | 1 |
|  |  | ETR | 226 | NA | 208 | 1,035 | 190 | 1,999 | 3* |
|  |  | WDI | 226 | NA | 208 | 1,256 | 190 | 1,365 | 1 |
|  |  | Deglac. | 226 | NA | 208 | 1,058 | 190 | 0,897 | 1 |
|  | SARerr  (480 km) | Tmin | 4 | NA | 22 | 15,93 | 40 | 24,278 | 1 |
|  |  | Tmax | 4 | NA | 22 | 16,616 | 40 | 24,034 | 1 |
|  |  | Pmin | 4 | NA | 22 | 16,766 | 40 | 24,038 | 1 |
|  |  | Pmax | 4 | NA | 22 | 17,764 | 40 | 32,276 | 3* |
|  |  | AET | 4 | NA | 22 | 15,887 | 40 | 24,717 | 1 |
|  |  | ETR | 4 | NA | 22 | 15,757 | 40 | 25,802 | 1 |
|  |  | WDI | 4 | NA | 22 | 16,475 | 40 | 22,168 | 1 |
|  |  | Deglac. | 4 | NA | 22 | 16,565 | 40 | 22,08 | 1 |
