# Supplementary material for: Relationships between climate and phylogenetic community structure of fossil pollen assemblages are not constant during the last deglaciation
Source: PLoS One. 2021 Jul 8;16(7):e0240957. doi: 10.1371/journal.pone.0240957 (PMC8266067; doi:10.1371/journal.pone.0240957)
Supplement: Attachment — Submitted filename: response2Reviews.pdf [file pone.0240957.s015.pdf]

Reviewer #2: Thank you for revising your manuscript, I think it has greatly improved (but there are still no line numbers).

> **Thank you for your review. We apologize for the lack of line numbers and for any inconvenience this caused. We did add line numbers to the document, but they seem to have been removed during the process of multiple authors editing between Word and Google Docs.**

Reviewer #3: This topic is interesting, the application prospect of this technique is also feasible, and the data in the manuscript is reliable

> **Thank you for your review.**

Reviewer #4: I see that the manuscript is at the R1 stage; I was not involved in the first round of reviews. The author responses to reviewers are thoughtful and the manuscript is written well. On a point raised by Reviewer1, I agree with the authors that discussing the implications of these analyses for pollen-based environmental interpretations is outside the scope of this analysis.

This paper carries value as one of the first mappings of phylogenetic tree data onto pollen data, which is non-trivial given the taxonomic ambiguities in pollen data. This paper makes the useful finding that PCS-climate relationships are not constant through time. All analytical and statistical methods seem basically sound to me; the pollen data handling relies on approaches developed in prior papers and the phylogenetic and spatial modeling analyses seem sound. The results are a bit difficult to interpret, but the paper does a good job of presenting conclusions that are relatively solid and noting caveats where appropriate.

> **Thank you for your review and your very helpful comments.**

A couple of thoughts that might be useful for the discussion and interpretation. First, most of the analyses and hypotheses are framed around assumptions that temperature is the primary environmental filter, and so assume that environmental filtering will be strongest in the north. However, in North America there are broadly two environmental gradients/filters: a north-south temperature filter and an east-west moisture filter. This second filter would help explain the pattern of phylogenetic clustering in the far western part of the study domain, where environmental filtering is strongest due to scarce water availability.

> **Thank you for this suggestion! We have edited our introduction and discussion to include the east-west moisture filter.**

Second, it seems striking to me that many of the overdispersed loci are in the central US, roughly at the continental ecotone between the Great Plains to the west and the mesic deciduous forests to the east. So, I wonder if some of the overdispersion comes at this physiognomic ecotone (grasses/herbs to the west, trees to the east) that I suspect also

represents a phylogenetic ecotone. If so, perhaps one could invoke ecotones as a previously underrecognized reason for phylogenetic overdispersion.

For the Caveats section, add the point that these analyses rely upon paleoclimatic simulations from Earth System Models, which carry known uncertainties and inaccuracies. If the ESMs are systematically wrong in some places, this could lead to some of the apparent shifts in PCS-climate relationships. Could also note that CO2 was much lower at the LGM and more diverse suites of megaherbivores were present, both of which might have altered plant-climate (and PCS-climate) relationships.

> **All great suggestions, thank you. We have edited the Caveats section to include these points.**

As an aside, two issues in the manuscript created unnecessary work for this volunteer peer reviewer. First, no line numbers were available, which makes it hard to precisely pinpoint comments. Second, the figure legends were scattered throughout the main text, while the figures were all at the end. This made it quite hard to find the matching legends and figures in my PDF. So, in the future, please put legends and figures all in one place. (I personally prefer all at the end of the ms., but others prefer embedding figures and text in the ms.)

> **We apologize for the lack of line numbers and the inconvenience this caused the reviewers. The lack of line numbers was pointed out to us in a previous round of review, and while we did add line numbers to the document, they seem to have been removed during the process of multiple authors editing between MS Word and Google Docs. That said, it was a failure on our part to not confirm line numbers were included on the submitted version.**
>
> **In terms of the figure legends being scattered throughout the text. We agree 100% that this is confusing. However, we were following the formatting requirements of the journal, which state "Figure captions must be inserted in the text of the manuscript, immediately following the paragraph in which the figure is first cited (read order). Do not include captions as part of the figure files themselves or submit them in a separate document."**

Other line-by-line comments:
For Figure 2, include in the panels a R2, p-value, or other measure of goodness of fit or significance.

> **We have added R^2 and p-values in Figure 2. Note, however, that we did not include them in the panels since there is just one R^2 and p-value for each of the three models we fitted. Those with changing intercepts (and slopes) through time are fit in a single model and R^2 and p-value applies to the whole dataset.**

Figure 4: what are the units for the slopes on the y-axis?

The units depend on the variable being plotted. We added a statement to the figure caption to clarify.

P6 ‘Extensive paleoecological time series’ – extensive in time and/or space? Clarify.

Sorry for any confusion, we feel the text that follows this statement clarifies that we mean extensive in both space and time:

“*Extensive paleoecological time series provide an opportunity to examine assemblage dynamics across large spatial extents and extended periods of time as species responded to climatic events, ...*”

P6: Good statement of expectations at the bottom of P6. Note too that one might expect stronger environmental filtering in the semi-arid climates of the Great Plains region.

Thank you for this suggestion, we edited the text at the bottom of page 6 as follows:

“*We expected to find a significant effect of climate on PCS of pollen assemblages, with phylogenetically clustered communities in places and at times where environmental filtering should dominate, namely high latitudes, semi-arid regions like the Great Plains, and harsh LGM climates. In contrast, we expect phylogenetically overdispersed communities in places and at times where the role of environmental filtering should be reduced, such as low latitudes, moist regions like the Appalachians, and benign present-day climates.*”

P7: This review of Ambrosia is a bit unclear and also omits Ambrosia’s resemblance to Iva.

Apologies for the lack of clarity. We edited the text as follows:

“*Additionally, Ambrosia-type includes multiple genera in Asteraceae that have similar pollen morphology that are generally indistinguishable. Nonetheless, within the Asteraceae Iva and Xanthium are distinguished in the pollen dataset and were treated independently.*”

P12: The models all seem fine but the review of models 2 & 3 both invoke lags as a reason for changes in either the intercept of slope. I strongly recommend removing any mention of lags at this point, because there are multiple reasons for why PCS-climate relationships might change over time.

We invoked lags here in response to the previous round of review, but we agree that there are numerous reasons for PCS-climate relationships might change

overtime and mentioning only lags here is misleading. In favor of clarity, we decided to remove mention of lags despite our previous edits.

P14 Fig 2 legend – use the ‘Stable-Relationship’ model terminology when referring to the leftmost plots, for consistency with Methods.

Done - thanks for the suggestion.

P24 Capitalize Northern Hemisphere

Fixed - thank you.

P27 times periods -> time periods

Fixed - thank you.
